# Rnr1, but not Rnr3, facilitates the sustained telomerase-dependent elongation of telomeres

**DOI:** 10.1371/journal.pgen.1007082

**Published:** 2017-10-25

**Authors:** André Maicher, Inbal Gazy, Sushma Sharma, Lisette Marjavaara, Gilad Grinberg, Keren Shemesh, Andrei Chabes, Martin Kupiec

**Affiliations:** 1 Dept. of Molecular Microbiology & Biotechnology, Tel Aviv University, Ramat Aviv, Tel Aviv, Israel; 2 Department of Medical Biochemistry and Biophysics, Umeå University, Umeå, Sweden; 3 Laboratory for Molecular Infection Medicine Sweden (MIMS), Umeå University, Umeå, Sweden; Chinese Academy of Sciences, CHINA

## Abstract

Ribonucleotide reductase (RNR) provides the precursors for the generation of dNTPs, which are required for DNA synthesis and repair. Here, we investigated the function of the major RNR subunits Rnr1 and Rnr3 in telomere elongation in budding yeast. We show that Rnr1 is essential for the sustained elongation of short telomeres by telomerase. In the absence of Rnr1, cells harbor very short, but functional, telomeres, which cannot become elongated by increased telomerase activity or by tethering of telomerase to telomeres. Furthermore, we demonstrate that Rnr1 function is critical to prevent an early onset of replicative senescence and premature survivor formation in telomerase-negative cells but dispensable for telomere elongation by Homology-Directed-Repair. Our results suggest that telomerase has a "basal activity" mode that is sufficient to compensate for the “end-replication-problem” and does not require the presence of Rnr1 and a different "sustained activity" mode necessary for the elongation of short telomeres, which requires an upregulation of dNTP levels and dGTP ratios specifically through Rnr1 function. By analyzing telomere length and dNTP levels in different mutants showing changes in RNR complex composition and activity we provide evidence that the Mec1^ATR^ checkpoint protein promotes telomere elongation by increasing both dNTP levels and dGTP ratios through Rnr1 upregulation in a mechanism that cannot be replaced by its homolog Rnr3.

## Author summary

Telomeres protect the ends of eukaryotic chromosomes and as such determine the replicative capacity of a cell. In budding yeast and approximately 80% of human tumors the enzyme telomerase maintains telomere length by adding newly synthesized repeats to telomeres using dNTPs generated by Ribonucleotide reductase (RNR) complexes. Similarly, telomerase activity can restore telomere length after more severe telomere shortenings that result from collapsed replication forks or lead to telomere over-elongation in the absence of negative regulators of telomerase. Here we provide evidence for two activity modes of telomerase that differentially depend on the major RNR subunit Rnr1. We demonstrate that telomere maintenance and a compensation of the "end-replication-problem" is possible under conditions where Rnr1 activity is absent but that a sustained elongation of short telomeres fully depends on Rnr1 activity. We show that the Rnr1-homolog, Rnr3, cannot compensate for this telomeric function of Rnr1 even when overall cellular dNTP values are restored.

## Introduction

Telomeres are nucleoprotein complexes that protect the linear ends of eukaryotic chromosomes from nucleolytic degradation and unwanted DNA repair activities [1–3]. Due to the "end-replication-problem" telomeres shorten with every cell division [2]. Once telomeres reach a critical length, with progressive number of chromosome replications they can no longer maintain their protective function and induce a permanent arrest of the cell cycle which has been referred to as replicative senescence [4–7]. Most cancer cells overcome this limitation of replicative capacity by upregulating telomerase, an enzyme whose expression is usually restricted to highly proliferative cells or early developmental stages [8,9]. A minority of tumors makes use of an Alternative-Lengthening of Telomere (ALT)-mechanism, which is based on homology-directed repair (HDR) to re-elongate their telomeres [10,11]. Budding yeasts constitutively express telomerase; when telomerase subunits are deleted, a senescence-like growth arrest is induced after approximately 60–80 cell divisions [4,12]. Similar to ALT-positive tumors, single yeast cells called “survivors” manage to overcome this arrest by using HDR-mechanisms for telomere elongation [13–15]. Genetic screens in yeast have uncovered a large number of genes responsible for the maintenance of a constant telomere length (*T*elomere *L*ength *M*aintenance or *TLM* genes) [16–18]. These genes affect diverse cellular pathways.

Ribonucleotide reductase (RNR) is a universally conserved enzyme that converts ribonucleotides to 2’-deoxyribonucleotides and provides the precursors for the generation of dNTPs, which are required for both DNA synthesis and repair [19–21]. Budding yeast RNR is a tetrameric complex, which in the absence of DNA damage consists mainly of a homodimer of the large Rnr1 subunits and a heterodimer of the small Rnr2 and Rnr4 subunits. The Rnr1 subunit harbors the catalytic site and two effector sites, which regulate substrate preference (specificity site) and overall enzymatic activity (activity site). A diferric tyrosyl radical is present in the small Rnr2 subunit and is essential for catalytic reduction of NDPs [19]. The Rnr2-paralog, Rnr4, plays a structural role by stabilizing the tetramer complex and is crucial for the assembly and activity of the Rnr2 co-factor [22,23]. RNR abundance and activity is largely regulated by the Mec1^ATR^ checkpoint, and its downstream effector kinases Rad53 and Dun1. When cells enter S-phase and in response to DNA damage Mec1^ATR^ upregulates RNR activity by inducing the degradation of the Rnr1 inhibitor Sml1 through its downstream kinase Dun1, thus ensuring the constant supply of dNTPs required for replication and repair [24,25]. Dun1 activation also results in the degradation of Dif1, leading to the release of the Rnr2 and Rnr4 subunits from the nucleus and thus promoting the formation of active RNR complexes in the cytoplasm [26,27]. Finally, Mec1^ATR^-Rad53 upregulates Ixr1, a positive transcription factor of *RNR1* [28]. An alternative large subunit, Rnr3, is usually expressed at very low levels under normal growth due to transcriptional repression by the Crt1/Rfx1 protein but is highly upregulated in response to DNA damage in a Mec1^ATR^ checkpoint dependent manner [29,30]. *In vitro*, Rnr3 is about 100 times less active than Rnr1 but increases its activity when associated with Rnr1, via a crosstalk between the activity site of Rnr1 and the Rnr3 catalytic site [31].

Changes in dNTP ratios that result from hypomorphic mutations in the large Rnr1 subunit have been proposed to affect the processivity of telomerase [32]. The concrete function of Rnr1 in telomere elongation, however, remained difficult to analyze since a full deletion of *RNR1* results in cell death or a severe growth defect. Furthermore, the investigation of a direct connection between Mec1^ATR^ and its downstream target RNR in telomere maintenance was impaired by the fact that survival of *mec1*Δ strains depends on dNTP level upregulation that is usually achieved by a co-deletion of the Rnr1-inhibitor *SML1* [33].

In this work we expressed an alternative Rnr3-containing RNR complex, which allowed us to study the consequences of a complete loss of Rnr1 function on telomere length regulation. Surprisingly, we found that Rnr1 activity not only promotes telomere maintenance by telomerase but is absolutely required for the sustained telomerase-dependent elongation of short telomeres. We demonstrate that a loss of Rnr1 activity specifically impairs the elongation of short telomeres by telomerase but leaves ALT-pathways unaffected. By using alternative ways to suppress the lethality of *mec1*Δ mutants we show that sustained telomere elongation by telomerase is promoted by the upregulation of Rnr1 activity, which also maintains high dGTP ratios. Furthermore, we demonstrate that telomere over-elongation is regulated by Mec1^ATR^-Dun1 pathway in an Rnr1-specific mechanism that cannot be replaced by an upregulation of Rnr3-produced dNTPs. As *rnr1*Δ mutants do maintain functional telomeres, but are unable to elongate them further, our results distinguish between two different telomerase activity “modes”, one involved in telomere maintenance, and another responsible for sustained elongation/repair.

## Results

### Rnr1 plays a role in the maintenance of telomeres, which cannot efficiently be replaced by its homolog Rnr3

**Fig 1:**
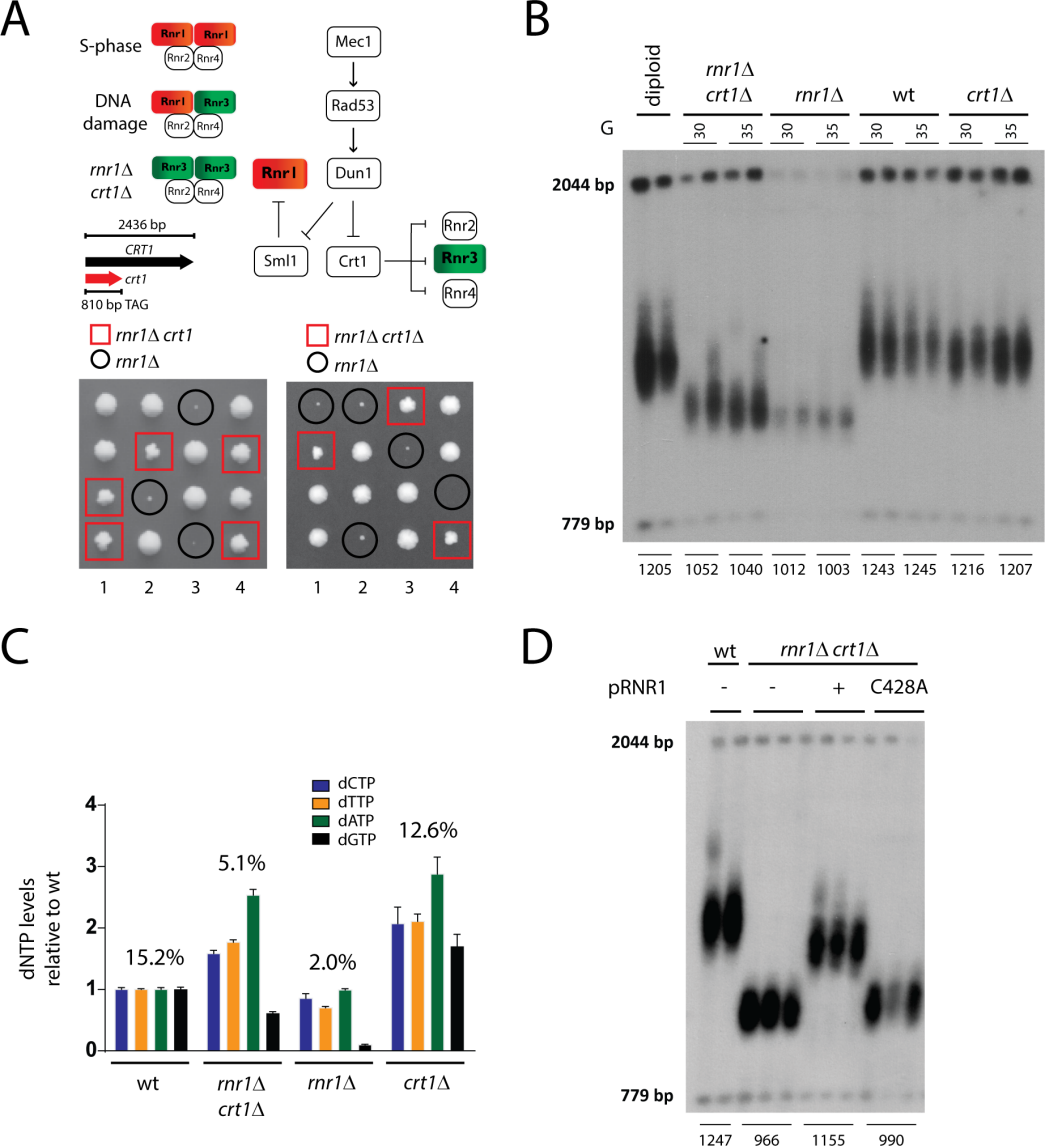
The loss of Rnr1 causes short telomeres that are stably maintained. **A)** Mec1^ATR^ regulates RNR activity and abundance via its downstream effector kinases Rad53 and Dun1. Dun1 phosphorylates Sml1 and releases its inhibitory effect on Rnr1 activity. Upon phosphorylation by Dun1, the repressive effect of Crt1 on Rnr2/3/4 transcription is abolished. The major RNR complex present in S-phase of the cell cycle consists of a tetramer containing two large Rnr1 subunits. Rnr3 is usually expressed at low levels but is upregulated in response to DNA damage. Sequencing of the *CRT1* gene identified a premature stop codon that overcame the slow growth defect of *rnr1*Δ mutants, indicating that an RNR complex containing only Rnr3 as large subunits can compensate for the loss of Rnr1 in terms of cell viability. Tetrad analysis of spores derived from *RNR1/rnr1*Δ *CRT1/crt1* (left) and *RNR1/rnr1*Δ *CRT1/crt1*Δ (right) heterozygous diploids indicate that the *crt1* suppressor mutation behaves as a single Mendelian trait and phenocopies the full deletion of *CRT1* in the suppression of impaired growth. For each heterozygous diploid four tetrads (1–4) have been analyzed by dissection. **B)** Telomere length analysis by Southern blotting. Following meiotic segregation heterozygous diploid *RNR1/rnr1*Δ *CRT1/crt1*Δ mutants have been dissected and the ability to maintain Y´ telomeres has been analyzed in the indicated spore colonies (2 biological replicates over 2 serial dilutions in liquid culture; approximately 30 and 35 generations after meiotic segregation). The telomere shortening of *rnr1*Δ *crt1*Δ double mutants resembles the one of *rnr1*Δ single mutants. **C)** dNTP pools and dGTP ratios (in %) were measured in the indicated strains. *rnr1*Δ mutants show lower dNTP levels and dGTP ratios when compared to wild type cells. *CRT1* mutation results in an elevation of dNTP levels of *rnr1*Δ mutants but dGTP levels remain below wild type levels. Mean values +/-SEM are indicated. All values are shown as fold change over the individual dNTP levels of the wild type (set to 1). n(wild type) = 9; n(*rnr1*Δ *crt1*Δ) = 3; n(*rnr1*Δ) = 3; n(*crt1*Δ) = 2. **D)** Telomere length analysis by Southern blotting showing that *rnr1*Δ *crt1*Δ mutant telomeres are short but stably maintained. The plasmid-born expression of wild type Rnr1 for 200 generations restored telomere length of *rnr1*Δ *crt1*Δ mutants to a large extent. The expression of the catalytic inactive Rnr1-C428A did not affect telomere length in *rnr1*Δ mutants. Represented are biological replicates of the indicated strains.

Budding yeast RNR is a tetrameric complex, which during S-phase consists mainly of a homodimer of Rnr1 and a heterodimer of Rnr2 and Rnr4 subunits (Fig 1A). The deletion of *RNR1* results in an impaired growth phenotype: in some yeast genetic backgrounds (e.g. W303) the loss of *RNR1* results in cell death, whereas in others, as the BY4741/2 background used here, cells grow slowly, forming extremely small colonies presumably because of a leaky transcription of the alternative Rnr3 subunit [32]. Spontaneous suppressors that allowed better growth of *rnr1*Δ mutants were found to contain a premature stop mutation in the *CRT1* gene that leads to the expression of a truncated Crt1 protein (Fig 1A). This mutation, as well as a full deletion of *CRT1*, efficiently suppressed the slow growth phenotype of *rnr1*Δ mutants after meiotic segregation and tetrad analysis (Fig 1A) and resulted in a clear elevation of Rnr3/4 protein levels (S1A Fig). We decided to make use of this system to study the effect of the absence of Rnr1 function on the maintenance of telomeres by telomerase. For this aim, an *RNR1/rnr1*Δ *CRT1/crt1*Δ double heterozygote strain was subjected to meiosis, and haploid progeny of all combinations was tested for the ability to maintain telomere length after approximately 30 and 35 cell divisions.

Consistent with previous results [32,34], we observed that the absence of Rnr1 resulted in pronounced telomere shortening (Fig 1B). To our surprise, however, an upregulation of Rnr3 expression (*CRT1* deletion) did not substantially alleviate the telomere shortenings of *rnr1*Δ mutants. Thus, in contrast to cell viability, which was largely rescued by an upregulation of RNR complexes containing only Rnr3 as large subunits (Fig 1A), the telomere length defect could not be restored in *rnr1*Δ mutants. We conclude that Rnr1-containing RNR complexes are more effective in the maintenance of telomere length than RNR complexes containing Rnr3.

Next we investigated how these changes in RNR complex composition affect overall cellular dNTP levels in these mutants (see S4 Table). Consistent with previous observations [32,34], cells lacking Rnr1 (wild type for *CRT1*), showed a reduction in cellular dNTP levels and particularly low dGTPs (Fig 1C). Interestingly, the upregulation of Rnr3-containing RNR complexes by a deletion of *CRT1* was sufficient to restore dATP,dTTP,dCTP but not dGTP levels above wild-type-levels in the absence of Rnr1 (Fig 1C). Thus, Rnr3-containing RNR complexes appear to be less efficient in the generation of dGTPs when compared to Rnr1-containing RNR complexes.

In order to determine potential long-term effects of the absence of Rnr1 on telomere length and to validate that the short telomere phenotype of *rnr1*Δ *crt1*Δ mutants is caused by a loss of the enzymatic function of Rnr1, we expressed wild type *RNR1* or a catalytically inactive version of Rnr1 (C428A) in *rnr1*Δ *crt1*Δ mutants for 200 generations. Notably, *rnr1*Δ *crt1*Δ mutants did not show any signs of replicative senescence but were able to maintain short telomeres even after 200 generations (Fig 1D). Thus, Rnr3-produced dNTPs are sufficient for compensation of the “end-replication-problem”. Expression of a wild type *RNR1* was able to complement the telomere defect of the *rnr1*Δ *crt1*Δ strain and a telomere length close to wild type (*crt1*Δ, compare Fig 1B) was restored after 200 generations (Fig 1D). In contrast, telomeres did not become re-elongated in *rnr1*Δ *crt1*Δ mutants expressing the catalytically inactive Rnr1-C428A allele. We also assessed potential long-term effects of the loss of Rnr3 function on telomere length. In contrast to a loss of Rnr1, a deletion of *RNR3* did not result in telomere shortening after 200 generations (S1B Fig).

Homology-directed repair (HDR) between short telomeres can contribute to their maintenance, in addition to telomerase [35]. In order to investigate whether HDR maintains the telomeres of *rnr1*Δ *crt1*Δ mutants, we generated heterozygous diploid *RNR1/rnr1*Δ *CRT1/crt1*Δ *RAD52/rad52*Δ mutants and assessed the telomere shortening in the derived spore colonies after meiotic segregation. The absence of *RAD52*, a gene essential for HDR, did not exacerbate the loss of telomeric sequences in *rnr1*Δ *crt1*Δ double mutants or lead to senescence (S1C and S1D Figs).

These results suggest that an Rnr1-specific activity of RNR is required to maintain wild type telomere length by telomerase. Rnr3-containing RNR complexes appear to function less efficiently in this telomere maintenance mechanism. As a consequence telomeres are eventually maintained at a shorter length in the absence of Rnr1 but can become re-elongated when wild type Rnr1 is re-introduced (Fig 1D).

### Mec1^ATR^ promotes the maintenance of wild type telomere length by maintaining high cellular dGTP ratios through Rnr1 upregulation

**Fig 2:**
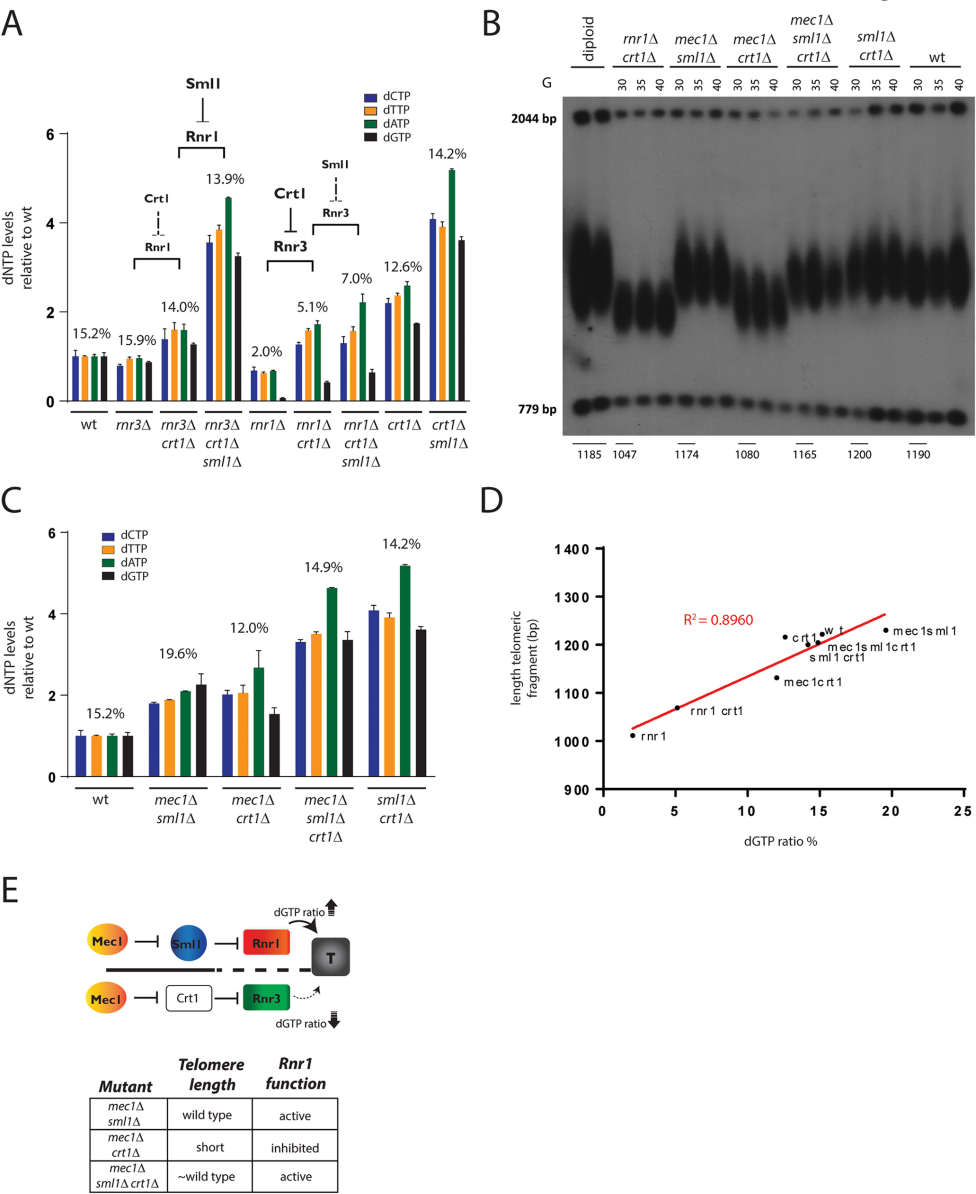
The Mec1^ATR^–checkpoint promotes telomere elongation by regulating Rnr1 through Sml1. **A)** Sml1 inhibits Rnr1 but not Rnr3 activity. dNTP pools and dGTP ratios (in %) were measured in the indicated strains. The co-deletion of *SML1* upregulates dNTP synthesis in *rnr3*Δ *crt1*Δ double mutants but not in *rnr1*Δ *crt1*Δ mutants. The co-deletion of *CRT1* increases dNTP synthesis in *rnr1*Δ but not substantially in *rnr3*Δ mutants. Mean values +/-SEM are indicated. All values are shown as fold change over the individual dNTP levels of the wild type (set to 1). The dNTP levels of *rnr1*Δ and *rnr1*Δ *crt1*Δ mutants were taken from the experiment in Fig 1C and are shown as a comparison. n(wt) = 2; n(*rnr3*Δ) = 2; n(*rnr3*Δ *crt1*Δ) = 2; n(*rnr3*Δ *crt1*Δ *sml1*Δ) = 2; n(*rnr1*Δ) = 3; n(*rnr1*Δ *crt1*Δ) = 3; n(*rnr1*Δ *crt1*Δ *sml1*Δ) = 2; n(*crt1*Δ) = 2; n(*crt1*Δ *sml1*Δ) = 2. **B)** Telomere length analysis by Southern blotting. Following meiotic segregation heterozygous diploid *MEC1/mec1*Δ *SML1/sml1*Δ *RNR1/rnr1*Δ *CRT1/crt1*Δ mutants were dissected and the ability to maintain telomeres was analyzed in the indicated spore colonies over 3 serial dilutions in liquid culture (generations 30G-40G). *mec1*Δ *crt1*Δ mutants show a short telomere length phenotype that resembles the one of *rnr1*Δ *crt1*Δ mutants. The short telomeres of *mec1*Δ *crt1*Δ could be rescued by a co-deletion of *SML1*. **C)** dNTP pools and dGTP ratios (in %) were measured in the indicated strains. *mec1*Δ *sml1*Δ, *mec1*Δ *sml1*Δ *crt1*Δ and *mec1*Δ *crt1*Δ mutants show dNTP levels above wild type. Mean values +/-SEM are indicated. All values are shown as fold change over the individual dNTP levels of the wild type which were taken from the experiment in **(A)** (set to 1). n(wt) = 2; n(*mec1*Δ *sml1*Δ) = 2; n(*mec1*Δ *sml1*Δ *crt1*Δ) = 2; n(*mec1*Δ *crt1*Δ) = 2. **D)** Telomere length has been analyzed as a function of dGTP ratios (dGTP levels relative to overall dNTP levels). An R-square test has been performed to investigate the goodness of fit. n(dNTPs/length): wt (9/3), *rnr1*Δ (3/2), *crt1*Δ (2/2), *rnr1*Δ *crt1*Δ (3/3), *sml1*Δ *crt1*Δ (2/1), *mec1*Δ *sml1*Δ (2/3), *mec1*Δ *crt1*Δ (2/3), *mec1*Δ *sml1*Δ *crt1*Δ (2/3). **E)** The Mec1^ATR^ checkpoint pathway promotes telomere elongation specifically via its regulation of Rnr1 activity through Sml1 degradation. High Rnr1 activity promotes telomere maintenance by keeping high cellular dGTP ratios. Shortening of telomere length correlates with Rnr1 inhibition or inactivity and therefore lower dGTP ratios that are produced by Rnr3.

When cells enter S-phase Mec1^ATR^ increases RNR activity by degrading the RNR inhibitor Sml1 (24). Sml1 is a known inhibitor of Rnr1 [24,25], but has also been reported to bind to Rnr3 [36]. Furthermore, in order to form the active RNR complex, Rnr1 and Rnr3 associate with the small subunits, which are regulated by Crt1 [30]. We were therefore interested in the question: to what extent Rnr1- and Rnr3- containing RNR complexes react to the degradation of Sml1 and Crt1? To this aim we quantified dNTP levels in *rnr3*Δ and *rnr1*Δ single mutants and compared them to *rnr3*Δ *crt1*Δ and *rnr1*Δ *crt1*Δ double mutants and *rnr3*Δ *crt1*Δ *sml1*Δ and *rnr1*Δ *crt1*Δ *sml1*Δ triple mutants (Fig 2A).

The deletion of *CRT1* resulted in an increased activity of Rnr3-containing RNR complexes (Fig 2A, compare *rnr1*Δ and *rnr1*Δ *crt1*Δ mutants) but only mildly affected Rnr1 activity (Fig 2A, compare *rnr3*Δ and *rnr3*Δ *crt1*Δ mutants). This small effect of a *CRT1* deletion on Rnr1-activity might be caused by the upregulation of Rnr2 and Rnr4, which might result in an increase in functional Rnr1-containing RNR complexes. Regarding the role of Sml1, we found that in contrast to Rnr1-containing RNR complexes (Fig 2A, compare *rnr3*Δ *crt1*Δ and *rnr3*Δ *crt1*Δ *sml1*Δ), Rnr3-containing RNR complexes do not substantially increase their dNTP synthesis rates in response to a deletion of *SML1* (Fig 2A, compare *rnr1*Δ *crt1*Δ and *rnr1*Δ *crt1*Δ *sml1*Δ). We conclude that the deletion of *CRT1* primarily increases the activity of Rnr3-containing RNR complexes whereas the absence of Sml1 upregulates Rnr1 activity (Fig 2A).

As mentioned before, the single deletion of *MEC1* results in cell lethality that can be suppressed by a co-deletion of *SML1*. *mec1*Δ *sml1*Δ double mutants show a telomere length that is similar to that of wild type cells [37]. We speculated that in these strains the function of Mec1 in telomere elongation might be masked by the co-deletion of *SML1*, which leads to the upregulation of Rnr1 activity independently of Mec1^ATR^. To test this possibility, heterozygous diploid *MEC1/mec1*Δ *SML1/sml1*Δ *RNR1/rnr1*Δ *CRT1/crt1*Δ cells (of wild type telomere length) were subjected to meiosis, dissected, and the ability to maintain telomeres was analyzed in the derived spores after 30–40 generations. We observed that *rnr1*Δ is synthetic lethal with *mec1*Δ even in the absence of *SML1 or/and CRT1* (S2A Fig). Thus, the epistatic relationships between *rnr1*Δ *crt1*Δ and *mec1*Δ *sml1*Δ in telomere maintenance could not directly be addressed. Consistent with previous results [30], we found that a deletion of *CRT1* suppresses the lethality of *mec1*Δ to a similar extent to that of a *SML1* deletion (S2A Fig). We decided to make use of these *mec1*Δ *crt1*Δ double mutants to investigate the effect of the absence of Mec1-mediated Sml1 degradation on telomere length.

Indeed, in agreement with the loss of an upregulation of Rnr1 activity by Sml1 degradation that promotes telomere elongation, the telomeres of *mec1*Δ *crt1*Δ mutants displayed a shorter length and resembled the telomeres of *rnr1*Δ *crt1*Δ mutants (Fig 2B). The co-deletion of *SML1* and resulting upregulation of Rnr1 activity (independent of Mec1) in *mec1*Δ *sml1*Δ *crt1*Δ mutants, however, resulted in an increased ability to maintain telomere length.

Next, we determined the cellular dNTP levels in the different *mec1*Δ mutants (Fig 2C). dNTP levels were upregulated above those of the wild type when *SML1* was deleted, even in the absence of Mec1^ATR^ (*mec1*Δ *sml1*Δ and *mec1*Δ *sml1*Δ *crt1*Δ). Interestingly, the deletion of *CRT1* was sufficient to upregulate the levels of all 4 dNTPs above wild type levels in *mec1*Δ mutants even in the presence of the active Sml1 inhibitor (*mec1*Δ *crt1*Δ). Thus, an overall cellular increase in dNTP levels produced by Rnr3 is not sufficient to restore telomere length when Mec1^ATR^ activity is lost and Rnr1 is inhibited by Sml1.

Changes in the ratios between dGTPs and the other dNTPs have been proposed to affect the processivity of telomerase leading to telomere length changes [32]. Since upregulation of Rnr3 (in strains deleted for *RNR1*) results in a relative dGTP deficit (Fig 1C), we asked to what extent the short telomere length of *rnr1*Δ, *rnr1*Δ *crt1*Δ mutants and *mec1*Δ *crt1*Δ mutants can be attributed to lower ratios of dGTPs. To this aim we analyzed telomere length in these mutants as a function of cellular dGTP ratios (dGTP levels relative to overall dNTP levels). Strikingly, we found that telomere length strongly correlated with dGTP ratios (Fig 2D; R^2^ = 0.8960) but not with overall cellular dGTP/dNTP levels (S2B Fig).

These results indicate that the Mec1^ATR^ checkpoint pathway promotes telomere maintenance by telomerase specifically by keeping high dGTP ratios through Rnr1 activation (Sml1 degradation) in S-phase. The upregulation of Rnr3 by a deletion of *CRT1* cannot replace this Rnr1 function in the absence of Mec1^ATR^ and eventually results in shorter telomeres due to a reduction in cellular dGTP ratios (Fig 2E).

### Telomerase- negative cells depend on Rnr1 activity to prevent an early onset of replicative senescence and premature survivor formation

Telomerase-negative yeast cells have recently been reported to show increased replicative stress at telomeres that can be suppressed by a co-deletion of the Rnr1 inhibitor *SML1* [38]. We were therefore interested in a potential role of Rnr1 in telomere maintenance that is independent of telomerase regulation.

**Fig 3:**
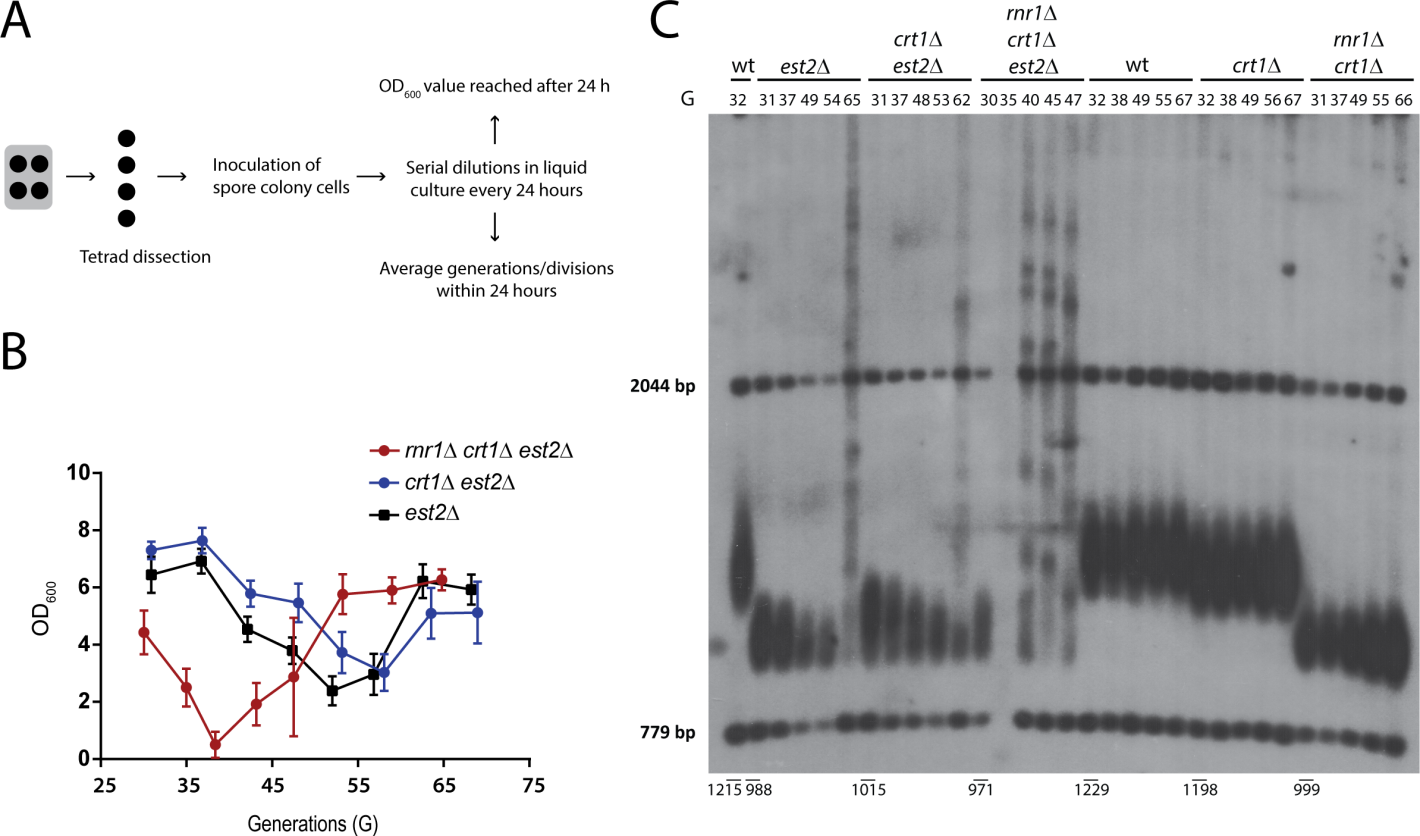
The loss of Rnr1 activity interferes with the maintenance of short telomeres and accelerates replicative senescence and survivor formation in telomerase-negative cells. **A)** Experimental approach: Following meiotic segregation and tetrad dissection spore colonies were diluted in liquid medium to an OD_600_ value of 0.02. After every 24h of growth at 30°C the optical cell density has been measured and cells were re-diluted to an OD_600_ of 0.02. The number of cell divisions/generations was calculated as log_2_ (OD_600_24h/0.02). **B)** Senescence curves reflecting the cell density reached after 24 hours of growth as a function of cell divisions (generations). In *rnr1*Δ *crt1*Δ *est2*Δ mutants the onset of replicative senescence and survivor formation is accelerated when compared to *crt1*Δ *est2*Δ and *est2*Δ controls. The number of cell divisions the spore colony went through before the first dilution has been estimated as 25 generations (starting point of the curves). Data is shown as mean +/- SEM (n = 3–5). **C)** Telomere length analysis by Southern blotting of one biological replicate of each genotype shown in **(B)**. Telomeres of *est2*Δ and *crt1*Δ *est2*Δ controls shortened progressively with ongoing generations (G) and showed a heterogeneous length indicative of survivor formation at 65 and 62 cell divisions, respectively. The telomeres of *rnr1*Δ *crt1*Δ *est2*Δ mutants could not be maintained after the first dilution and showed a heterogeneous length already at generation 40.

To this aim, the catalytic subunit of telomerase, *EST2*, was deleted in a heterozygous diploid *RNR1/rnr1*Δ *CRT1/crt1*Δ mutant and the rate of replicative senescence of *rnr1*Δ *crt1*Δ *est2*Δ spores was compared to that of isogenic *est2*Δ and *crt1*Δ *est2*Δ controls. Freshly dissected spore colonies were incubated in liquid YPD medium and re-diluted every 24 hours to an OD of 0.02. The cell density after 24 hours as a function of the number of cell divisions was used to visualize the onset of replicative senescence that is induced by telomere erosion (Fig 3A). As expected, *est2*Δ and *crt1*Δ *est2*Δ double mutants senesced after approximately 55–60 cell divisions in the absence of telomerase activity and eventually recovered from the growth arrest by forming survivors (Fig 3B). Interestingly, *rnr1*Δ *crt1*Δ *est2*Δ triple mutants showed a significant accelerated loss of viability and senesced at around generation 40, forming survivors prematurely (Fig 3B). In two out of five *rnr1*Δ *crt1*Δ *est2*Δ replicates survivors were already emerging in the first dilution suggesting that the loss of Rnr1 may have a synergistic effect on the promotion of HDR-mediated telomere lengthening in telomerase-negative cells (see S3A Fig for individual curves).

Of note, the loss of viability observed at early dilutions in *rnr1*Δ *crt1*Δ *est2*Δ triple mutants was not observed in telomerase-positive *rnr1*Δ *crt1*Δ cells, indicating that the senescence is telomere-related and not due to a checkpoint activation that is elicited by DNA damage elsewhere in the genome (S3B Fig). To validate these observations, we directly determined the telomere lengths of the re-diluted mutants by Southern blotting (Fig 3C) using the biological replicates that best described the average curves in Fig 3B. After the first dilution, telomeres of *rnr1*Δ *crt1*Δ *est2*Δ mutants showed a similar length to those of *est2*Δ and *crt1*Δ *est2*Δ mutants. With ongoing dilutions, however, the telomeric signals differed significantly. Whereas telomeres of *crt1*Δ *est2*Δ and *est2*Δ mutants continued to shorten progressively and only formed survivors after approximately 30 more generations, the telomeres of *rnr1*Δ *crt1*Δ *est2*Δ mutants showed a heterogeneous length indicative of survivor formation already after 10 more cell divisions (Fig 3C).

Overall these results suggest that Rnr1 activity not only promotes telomere length maintenance by telomerase but functions additionally in a mechanism that is crucial for the maintenance of short telomeres in the absence of telomerase. The fact that *rnr1*Δ *crt1*Δ *est2*Δ mutants formed survivors prematurely shows that their telomeres can efficiently be extended by HDR-dependent telomere elongation mechanisms that do not require Rnr1 activity.

### A sustained elongation of short telomeres by telomerase requires Rnr1 activity

**Fig 4:**
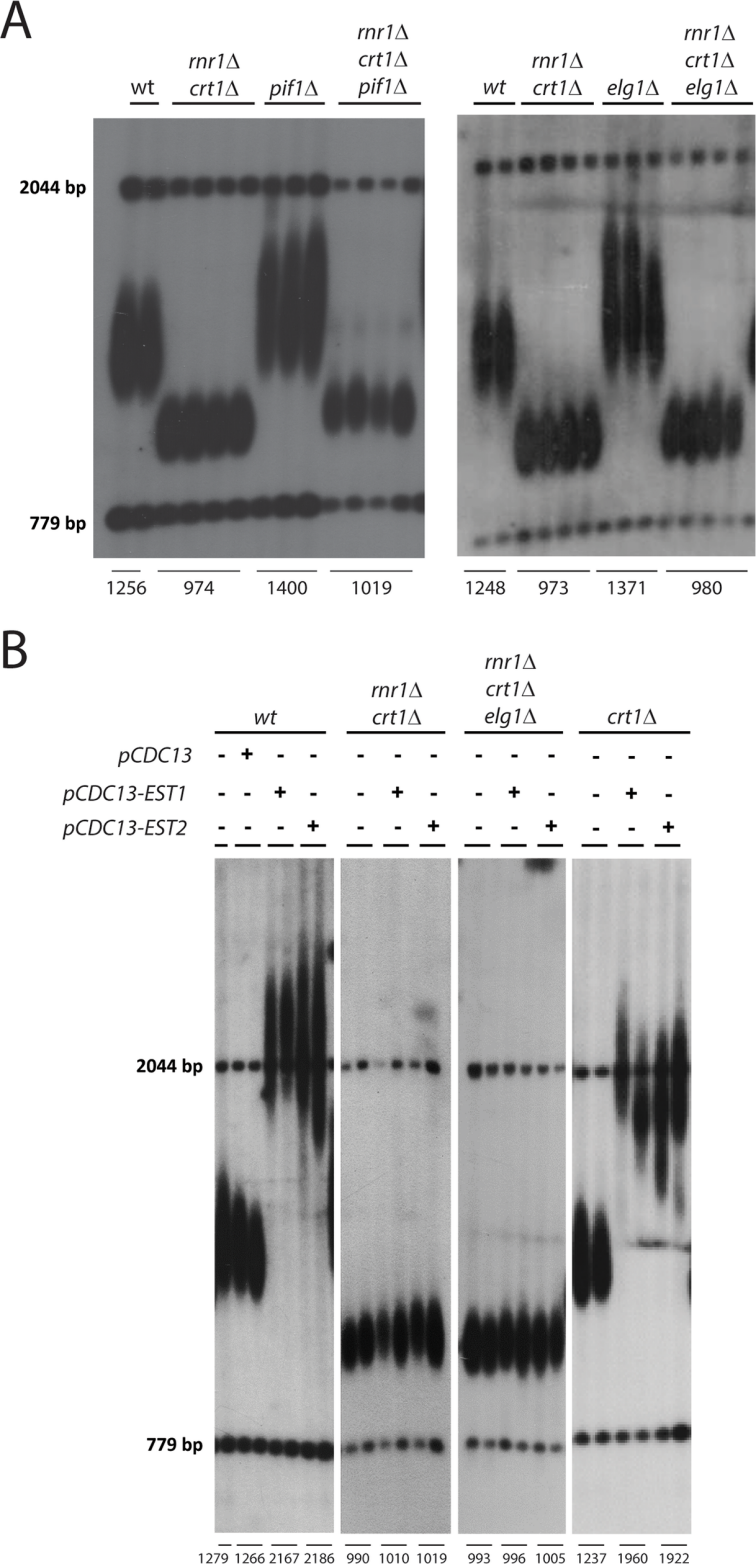
In the absence of Rnr1 telomeres cannot become elongated by increased telomerase activity. **A)** Telomere length analysis by Southern blotting shows that mutations of the long *TLM* genes *PIF1* or *ELG1* cause telomere elongation when introduced in a wild type background but do not affect the short telomere length of *rnr1*Δ *crt1*Δ mutants. Telomere blots were performed approximately 200 generations after the long *tlm* mutation has been introduced. Represented are biological replicates of the indicated strains. **B)** Telomere length analysis by Southern blotting of cells expressing Cdc13-Est1 and Cdc13-Est2. Expression of the fused proteins results in telomere elongation in wild type cells and *crt1*Δ mutants but not in *rnr1*Δ *crt1*Δ mutants or *rnr1*Δ *crt1*Δ *elg1*Δ mutants. A plasmid expressing Cdc13 was used as a control and did not cause elongation of wild type cell telomeres. Represented are biological replicates of the indicated strains approximately 200 generations after transformation of the indicated plasmid.

Mutations in Rnr1 that reduce the ratio of dGTP synthesis have been reported to negatively affect the processivity of telomerase [32]. Since we observed lower dGTP ratios in *rnr1*Δ *crt1*Δ mutants (Figs 1C and 2D) we next asked whether an increase in telomerase activity would compensate for the complete loss of Rnr1 function at telomeres. Deletions of a number of yeast genes have been shown in the past to lead to telomere over-elongation [hereafter referred as “long *tlm* (*t*elomere *l*ength *m*aintenance) mutants”] in a telomerase-dependent manner. We tested whether deleting any of these genes would restore normal telomere size in *rnr1*Δ *crt1*Δ mutants. Pif1 acts as a negative regulator of telomere length by inhibiting telomerase processivity and therefore limits the length of the telomeric sequence that is added in a single telomerase reaction [39]. Elg1 is an RFC-like complex, which has been implicated in the coupling of semiconservative replication and telomerase activity [40,41] and acts in a different pathway of telomere elongation from Pif1 [42]. As expected, deletion of these genes in a wild type *RNR1* background led to telomere elongation (Fig 4A). In stark contrast, telomere length did not change when these long *tlm* mutations were introduced into *rnr1*Δ *crt1*Δ cells. In fact, telomeres remained as short as in the *rnr1*Δ *crt1*Δ double mutant controls even after 200 generations (Fig 4A). We tested several additional long *tlm* mutations for their ability to elongate the short telomeres of *rnr1*Δ *crt1*Δ mutants and found that none of them was capable of causing telomere elongation (S4 Fig). We next tried to force elongation in *rnr1*Δ *crt1*Δ mutants by expression of a fusion protein between the telomere-binding protein Cdc13 and either the regulatory subunit of telomerase, Est1, or the catalytic subunit, Est2. These fusion proteins result in a permanent recruitment of telomerase to telomeres and in telomere elongation in wild type cells [43] (Fig 4B). Strikingly, telomeres of *rnr1*Δ *crt1*Δ and *rnr1*Δ *crt1*Δ *elg1*Δ mutants did not become elongated by expression of either of these fusion proteins (Fig 4B). These results indicate that genetic alterations that elevate the activity or processivity of telomerase in the presence of Rnr1 are unable to do so in the absence of Rnr1. Thus, *rnr1*Δ *crt1*Δ mutants are proficient in coping with the "end-replication-problem", but unable to extend short telomeres in a sustained way.

### A Mec1^ATR^–Dun1 mediated upregulation of Rnr1-produced dNTPs promotes telomere (over-)elongation

**Fig 5:**
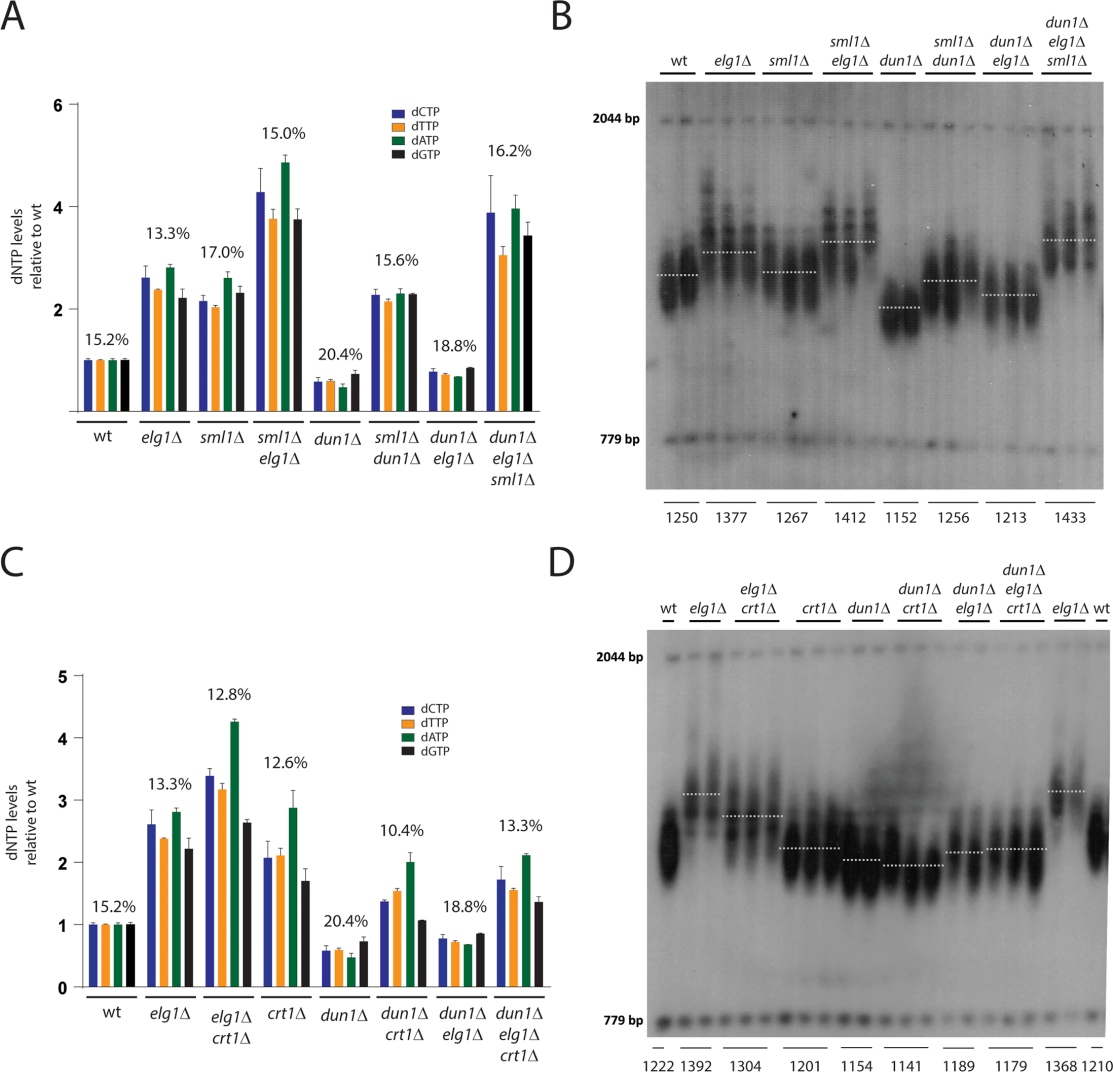
The Mec1^ATR^-Dun1 pathway promotes telomere over-elongation via Rnr1 activation through Sml1 degradation. **A)** dNTP pools and dGTP ratios (in %) were measured in the indicated strains. *dun1*Δ mutants show low dNTP levels, which can be elevated above wild type levels by a co-deletion of *SML1*. In *sml1*Δ *elg1*Δ *dun1*Δ mutants dNTP levels are elevated approximately 4 fold relative to wild type. Mean values +/-SEM are indicated. All values are shown as fold change over the individual dNTP levels of the wild type which were taken from the experiment in Fig 1C (set to 1). n(wt) = 9; n(*elg1*Δ) = 2; n(*sml1*Δ) = 2; n*(sml1*Δ *elg1*Δ) = 2; n(*dun1*Δ) = 2; n(*sml1*Δ *dun1*Δ) = 2; n (*dun1*Δ *elg1*Δ) = 2; n(*sml1*Δ *dun1*Δ *elg1*Δ) = 2. **B)** Telomere length analysis by Southern blotting. A deletion of *DUN1* results in short telomeres, which only slightly become elongated by a co-deletion of the long *TLM* gene *ELG1*. The simultaneous deletion of *SML1* restores telomere length in *dun1*Δ mutants and rescues the over-elongation of telomeres in *dun1*Δ *elg1*Δ double mutants. Represented are biological replicates of the indicated strains after approximately 200 generations. **C)** dNTP pools and dGTP ratios (in %) were measured in the indicated strains. *dun1*Δ mutants show low dNTP levels, which can be elevated above wild type levels by a deletion of *CRT1* or simultaneous deletions of *CRT1* and *ELG1*. Mean values +/-SEM are indicated. All values are shown as fold change over the individual dNTP levels of the wild type which were taken from the experiment in Fig 1C (set to 1). The dNTP levels of *elg1*Δ and *dun1*Δ mutants were taken from the experiment in **(A)** and are shown as a comparison. n(wt) = 9; n(*elg1*Δ) = 2; n(*elg1*Δ *crt1*Δ) = 2; n*(crt1*Δ) = 2; n(*dun1*Δ) = 2; n(*dun1*Δ *crt1*Δ) = 2; n(*dun1*Δ *elg1*Δ) = 2; n(*dun1*Δ *elg1*Δ *crt1*Δ) = 2. **D)** Telomere length analysis by Southern blotting. A co-deletion of *CRT1* does not restore telomere length in *dun1*Δ mutants nor does it rescue the over-elongation of telomeres in *dun1*Δ *elg1*Δ double mutants. Represented are biological replicates of the indicated strains after approximately 200 generations.

Our results so far highlighted a critical function of Rnr1-containing RNR complexes in the sustained elongation of short telomeres by telomerase (Fig 4). In order to determine whether this function of Rnr1 is regulated by the Mec1^ATR^ checkpoint we deleted the Dun1 kinase, which acts downstream of Mec1^ATR^ in the regulation of RNR activity (Fig 1A) [24,25,30]. Consistent with previous results [17,32,44] *dun1*Δ mutants showed reduced dNTP levels and short telomeres, which were suppressed by a co-deletion of the Rnr1 inhibitor *SML1* (Figs 5A and 5B). To address whether reduced dNTP levels also result in telomere shortenings in long *tlm* mutants we next combined the *dun1*Δ mutation with deletions of genes that negatively regulate telomerase-dependent telomere elongation. Indeed, the deletion of *ELG1* only caused a mild telomere elongation in the absence of Dun1 suggesting that RNR activity contributes to telomere over-elongation in long *tlm* mutants (Fig 5B).

We have shown before that the upregulation of Rnr1 promotes telomere elongation in *mec1*Δ *sml1*Δ and *mec1*Δ *sml1*Δ *crt1*Δ mutants whereas the upregulation of Rnr3 is not sufficient to restore wild type telomere length in *mec1*Δ *crt1*Δ mutants (Fig 2B). In order to determine whether the same applies for long *tlm* mutants, we deleted *SML1* or *CRT1* to elevate the activity/level of Rnr1 and Rnr3-containing RNR complexes in *elg1*Δ *dun1*Δ mutants. Interestingly, we found that the deletion of *SML1* and therefore Rnr1 upregulation fully rescued the telomere elongation defect of *elg1*Δ *dun1*Δ mutants (Fig 5B). Consistent with our results using a *mec1*Δ *crt1*Δ mutant (Figs 2B and 2C) the co-deletion of *CRT1* did not affect the short telomere length of *dun1*Δ mutants, despite the fact that it was able to elevate cellular dNTP levels above those of the wild type (Figs 5C and 5D). Thus, the short telomere phenotypes in the absence of Dun1 and Mec1 activity does not correlate with a general reduction of dNTPs but specifically with a reduction in Rnr1 activity. Moreover, in stark contrast to *SML1* deletion, a deletion of *CRT1* did not counteract the suppression of telomere elongation in *elg1*Δ mutants by a *DUN1* co-deletion even though total levels of dNTPs were clearly upregulated in *dun1*Δ *elg1*Δ *crt1*Δ mutants (Figs 5C and 5D). These results indicate that Dun1 promotes telomere elongation by a mechanism that specifically involves the upregulation of Rnr1 activity through Sml1 degradation. This activity of Rnr1 cannot be replaced by that of Rnr3. The positive effect of Rnr1 activity on sustained telomere elongation was not restricted to an *elg1*Δ strain. Indeed, several other long *tlm* mutations, which affected telomere length in a pathway different from that of *elg1*Δ (as indicated by the additive effects of the double mutation on telomere length) showed impaired telomere elongation in a *dun1*Δ mutant background that could be restored by *SML1* but not *CRT1* deletion (S5A Fig). Thus, an upregulation of Rnr1 activity through Dun1 seems to have a general positive effect on telomere over-elongation regardless of the original reason for telomere over-elongation in the respective long *tlm* mutant. Of note, in contrast to *rnr1*Δ *crt1*Δ mutants where telomerase-tethering did not result in any telomere elongation (Fig 4B), the expression of Cdc13-Est1 or Cdc13-Est2 fusion proteins caused elongation in *dun1*Δ *crt1*Δ mutants even though at a reduced level compared to wild type (S5B Fig). We conclude that, whereas Rnr1 is absolutely required for sustained elongation of telomeres, Dun1 promotes the sustained telomere elongation through Rnr1 activation but is not absolutely essential for it.

We have demonstrated that the short telomere length in the absence of Rnr1 or Mec1^ATR^ correlates with a reduction in dGTP ratios (Fig 2D). Surprisingly, we found that *elg1*Δ single mutants despite showing over-elongated telomeres were characterized by a slight reduction in dGTP ratios (~13%) presumably caused by an upregulation of Rnr3, as indicated by a largely epistatic effect of deleting *CRT1* in the *elg1*Δ strain on dNTP levels and ratios (Fig 5C). Thus, slightly reduced overall dGTP ratios (possibly above a certain threshold) seem to be sufficient for sustained telomere over-elongation. Consistent with these results, dGTP ratios and telomere length of *elg1*Δ *crt1*Δ double mutants resembled those of *elg1*Δ mutants (Figs 5C and 5D). Interestingly, we also observed that *dun1*Δ *elg1*Δ mutants showed higher dGTP ratios (~18%) but reduced telomere length compared to wild type. Thus, an increase in dGTP ratios alone does not correlate with telomere elongation when overall dNTP levels are limiting due to a loss of Dun1 activity.

Overall these results suggest that neither an increase in dGTP levels (*dun1*Δ *elg1*Δ *crt1*Δ) nor an increase in dGTP ratios alone (*dun1*Δ *elg1*Δ) is sufficient to promote telomere over-elongation in long *tlm* mutants in the absence of Rnr1 activation (Figs 5C and 5D). We therefore propose that the upregulation of Rnr1 by Dun1 promotes the sustained elongation of telomeres in 2 ways—by increasing overall dNTP levels and simultaneously keeping dGTP ratios at levels close to wild type. The fact that *dun1*Δ *elg1*Δ *crt1*Δ mutant telomeres remained impaired in telomere elongation despite having ratios sufficient for telomere over-elongation (~13%; compare dGTP ratios and telomere length of *elg1*Δ *crt1*Δ mutants in Figs 5C and 5D) and dNTP levels above wild-type-levels opens the possibility of an additional level of regulation of sustained telomere elongation that distinguishes between Rnr1- and Rnr3-produced dNTPs.

## Discussion

By suppressing the growth defect of *rnr1*Δ mutants through an upregulation of the Rnr1-homolog, Rnr3 (via *CRT1* deletion), we demonstrate that Rnr1 function not only promotes the maintenance of wild type telomere length but is also essential for telomere over-elongation by telomerase. Indeed, long *tlm* mutants, which exhibit elongated telomeres in a wild type background [16–18], were unable to elongate their telomeres in the absence of Rnr1 (Fig 4A and S4 Fig). Strikingly, even forced recruitment of telomerase did not result in telomere lengthening when Rnr1 was absent (Fig 4B).

**Fig 6:**
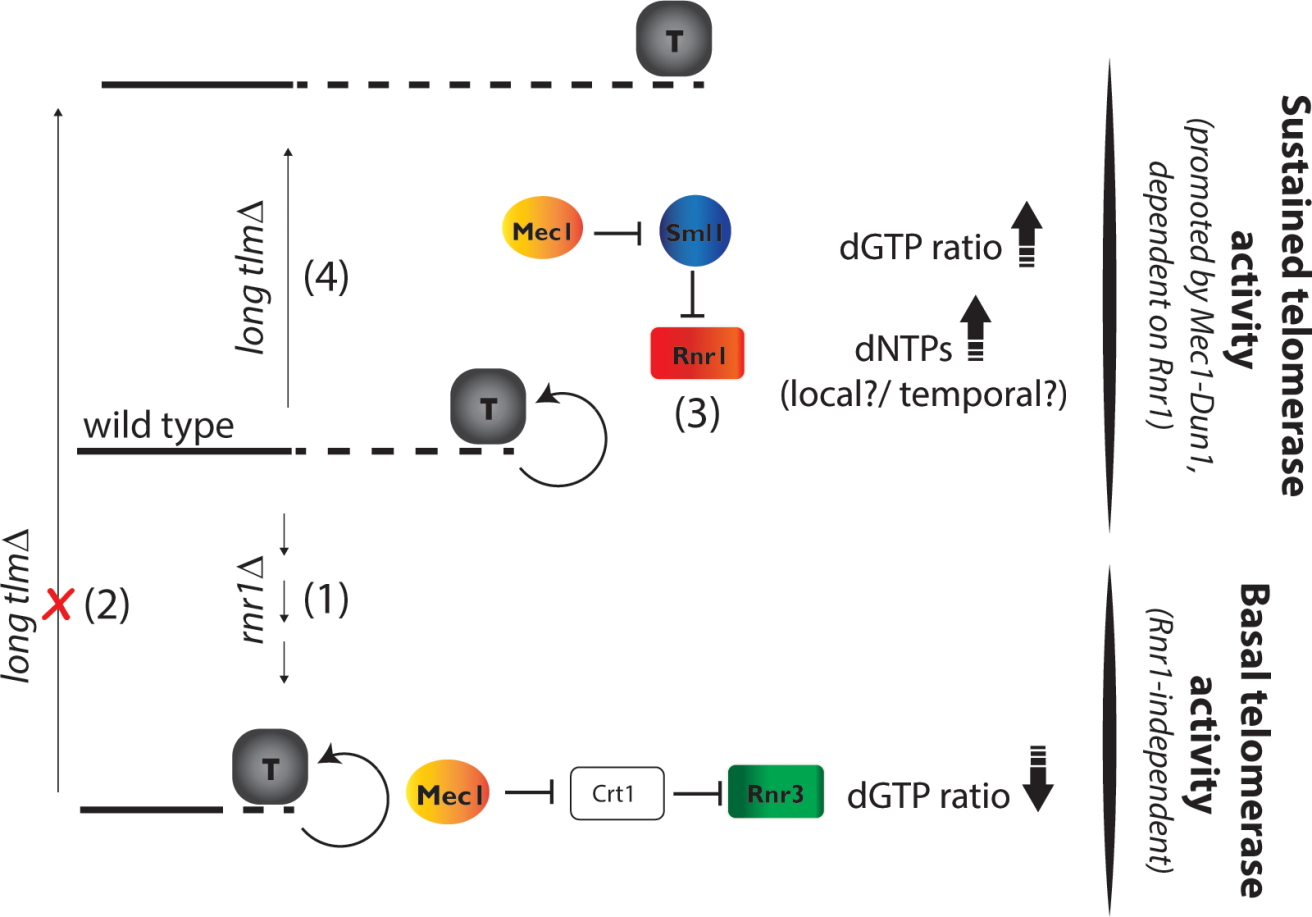
Model: Rnr1 but not Rnr3 provides telomerase with sustained activity. Rnr1 is essential for sustained telomerase-dependent elongation of telomeres. In the absence of Rnr1 (1) telomeres shorten and the expression of the alternative large subunit, Rnr3, only allows a basal activity of telomerase due to reduced dGTP ratios. This activity is sufficient to compensate for the "end replication problem" but not for re-elongation of the short telomeres even when long *TLM* genes become mutated (2). In wild type cells Mec1^ATR^ upregulates Rnr1 activity through Sml1 degradation and therefore ensures that telomeres are maintained at a wild type length by keeping high dGTP ratios and supplying dNTPs (3). Alike, in long *tlm* mutants telomere over-elongation is promoted by Rnr1 activation through Mec1^ATR^ (4). In both cases Rnr3 expression cannot compensate for this function of Rnr1 suggesting that Rnr1-produced dNTPs are provided in a local or temporal manner to promote telomere elongation. Stalled replication forks at telomeres (not displayed) might promote telomerase activity by triggering the S-phase checkpoint, thus maintaining high Rnr1 activity when telomeres become replicated.

The fact that *rnr1*Δ *crt1*Δ mutants are able to deal with the “end-replication-problem”, maintaining functional (albeit short) telomeres, but are unable to re-elongate their short telomeres lets us propose the existence of two different activity modes for telomerase, which we call “basal activity” and “sustained activity” (Fig 6). Whereas the first mode allows the maintenance of functional telomeres and compensation of the “end-replication-problem” in the absence of Rnr1, it is unable to provide extensive telomerase activity.

A previous report suggested a role of Mec1^ATR^ in telomere elongation, as hypomorhic *mec1* mutant alleles caused shorter telomeres [32]. Similarly, the short telomeres of *dun1*Δ mutants have been attributed to reduced dNTPs as both phenotypes could be suppressed by deleting the Rnr1 inhibitor *SML1* [32,44]

Here we have investigated the telomere length phenotype of a full deletion of *MEC1* and deciphered the role of the Mec1-Dun1 downstream targets Rnr1 and Rnr3 in telomere length regulation. Surprisingly we found that Mec1-Dun1 regulates telomere length maintenance specifically by Rnr1 in a mechanism that cannot be efficiently replaced by Rnr3. Indeed, *mec1*Δ mutants whose lethality was suppressed by upregulating Rnr3 (*CRT1* deletion) were impaired in telomere length maintenance due to the persistence of Rnr1 inhibition by Sml1, despite the upregulation of overall Rnr3-produced dNTP levels above those of wild type (Figs 2B and 2C). The same was true when Rnr1 activity was reduced by a *DUN1* deletion (Figs 5C and 5D). Thus, the short telomere phenotype of *mec1*Δ and *dun1*Δ mutants are not primarily caused by a general reduction in cellular dNTP levels but rather correlate specifically with a reduction in Rnr1 activity.

Consistent results were obtained when *DUN1* was deleted in long *tlm* mutants since the full capacity to over-elongate telomeres in *dun1*Δ *elg1*Δ mutants could only be restored when *SML1* but not when *CRT1* was co-deleted (Figs 5B and 5D). Thus, we conclude that similar to the maintenance of wild-type-length telomeres (Fig 2B) the Mec1-Dun1 pathway promotes telomere over-elongation in long *tlm* mutants specifically in an Rnr1-dependent mechanism. Why can’t Rnr3 compensate for this telomeric function of Rnr1? Previous experiments using hypomorphic *rnr1* mutants attributed their short telomere phenotype to changes in dNTP ratios and, in particular, reduced dGTP levels relative to other dNTPs [32]. Indeed, consistent with this idea, we found that the short telomere length in *rnr1*Δ and *mec1*Δ *crt1*Δ mutants correlated with reduced dGTP ratios (Figs 2D and 2E**).** Similarly, a reduction in dGTP ratios that was induced by a *CRT1* deletion correlated with slightly shorter telomeres (Fig 1B) and a small reduction in telomere over-elongation after telomerase tethering (Fig 4B). Of note, high dGTP ratios alone, however, appear to be not sufficient for sustained telomere elongation as *dun1*Δ mutants were characterized by elevated dGTP ratios and yet contained short telomeres (Figs 5A and 5B).

Similarly, when *ELG1* was deleted in *dun1*Δ mutants, telomere elongation remained reduced despite the fact that dGTP ratios where clearly above wild-type-levels (Figs 5A and 5B). This indicates that Rnr1 promotes telomere maintenance and over-elongation in long *tlm* mutants not only by keeping constant dGTP ratios but also by upregulating overall dNTP levels (Fig 6). Nevertheless, Rnr3-produced dNTPs cannot replace Rnr1-produced dNTPs in this mechanism, as shown by the inability of a *CRT1* deletion to restore telomere length in *dun1*Δ single and *dun1*Δ *elg1*Δ double mutants (Figs 5C and 5D).

Overall these results suggest an additional level of regulation that distinguishes between Rnr1 and Rnr3-produced dNTPs during telomerase-dependent telomere elongation (Fig 6). At least two different explanations are conceivable:

(I) Since Sml1 specifically inhibits Rnr1 (Fig 2A) and becomes degraded in a Mec1^ATR^-checkpoint-dependent manner in S-phase [24,25] a cell cycle regulated production of Rnr1-produced dNTPs might be crucial for efficient telomere elongation by telomerase. (II) A local (nuclear or perinuclear) activation of Rnr1 through Sml1 degradation might provide sufficient dNTPs for telomere elongation by telomerase. Indeed, even though Rnr1 has been shown to predominantly localize to the cytoplasm [45], an increased nuclear localization has been reported in response to replicative stress caused by a treatment with the RNR inhibitor hydroxyurea [46]. Human RNR can also be localized to the nucleus [47].

Of note, we found that *dun1*Δ mutant telomeres (compared to Dun1 positive cells) were impaired in over-elongation when long *TLM* genes were deleted but still could become elongated to some extent (Fig 5B and S5A Fig). Similarly, telomerase tethering was able to elongate telomeres of *dun1*Δ *crt1*Δ double mutants (albeit at a reduced rate) (S5B Fig). This opens the possibility that there is a threshold of dGTP ratios that is required for the ability to elongate telomeres in the “sustained telomere elongation” mode, which is ensured by the presence of Rnr1 but does not fully depend on its upregulation through Dun1. When dGTP ratios however drop due to a complete loss of Rnr1 function, telomeres can only be maintained by a basal activity using Rnr3-produced dNTPs (Fig 6).

The concrete molecular mechanism through which Rnr1-produced dNTPs and changes in dGTP ratios affect telomerase dependent telomere elongation remains to be determined. The ability to processively add telomeric DNA depends on two types of telomerase-movements which have been referred as type I and type II translocation [48]. Whereas the first determines the ability to add nucleotides with a simultaneous movement of the RNA-DNA duplex relative to the active site, the second involves an unpairing of the RNA-DNA hybrid that is followed by a re-alignment of telomerase to allow additional cycles of repeat addition. Studies using hypomorphic *rnr1* mutants showed that the nucleotide addition processivity (type I translocation) of telomerase correlates with changes in dGTP ratios *in vivo* [32]. Indeed, we find that a deletion of *PIF1* did not result in telomere elongation in the absence of Rnr1 (Fig 4A) opening the possibility that Rnr1 function is a pre-requisite for elevated nucleotide addition processivity [39]. Interestingly, however, both types of translocations have been shown to be promoted by increasing dGTP concentrations in *in vitro* experiments using budding yeast telomerase [49–51]. Also, elevated dGTP concentrations have been shown to increase the processivity of recombinant *Tetrahymena* and human telomerase *in vitro* [50,52,53]. Further biochemical experiments are required to address the question of whether *rnr1*Δ *crt1*Δ mutants are defective in nucleotide addition and/or repeat addition processivity.

Interestingly, upon telomerase ablation, an activation of the replication checkpoint adaptor Mrc1 and its downstream effectors Dun1 and Rad53 have been reported [54]. It therefore appears possible that there is a replication checkpoint-mediated upregulation of Rnr1 activity that facilitates fork progression through the short telomeric tracts and simultaneously promotes their telomerase-dependent extension. Consistent with this idea we found that the absence of Rnr1 accelerated the onset of replicative senescence and promoted survivor formation in telomerase negative cells (Figs 3B and 3C). This may be explained by replication fork collapses in *rnr1Δ crt1Δ est2Δ* mutants that become restarted by HDR, leading to the premature formation of survivors in the absence of both telomerase and Rnr1. Indeed, a checkpoint-dependent induction of RNR has recently been shown to promote the restart of stalled replication forks in response to replicative stress [55]. The fact that telomerase-negative *rnr1*Δ *crt1*Δ mutants are capable to efficiently elongate their telomeres by HDR further indicates that telomerase-dependent telomere elongation is more sensitive to a loss of Rnr1 activity than HDR-mediated elongation mechanisms.

Human RNR complexes consist of hetero-oligomers of two large alpha subunits, RRM1, and two small beta subunits, RRM2 or p53R2, which become upregulated in response to DNA damage [56]. Interestingly, RNR has been shown to localize to sites of DNA damage in human cells [47]. Furthermore, stalled replication forks have recently been shown to increase telomerase recruitment in an ATR-dependent manner in human cells [57]. It will therefore be interesting to determine whether the increased localization of telomerase to telomeres in response to stalled replication forks is caused by an S-phase checkpoint-mediated upregulation of RNR activity that promotes the sustained elongation of telomeres in higher eukaryotes. Furthermore, our results in budding yeast suggest that telomerase-positive cancer cells may be more sensitive to RRM1/RRM2 inhibition than ALT-positive cancer cells.

## Materials and methods

### Strains, primers and plasmids

All strains were derived from the BY4741/2 (*MAT a/alpha his3*Δ*0 leu2*Δ*0 ura3*Δ*0*) background. Detailed genotypes, primers and plasmids used in this study are listed in the S1–S3 Tables.

### Southern teloblots

Teloblots were carried out as previously described [18]. The telomeric probe was generated using the primers Y' element fwd / rev in a PCR reaction on genomic DNA of wild type cells (see S2 Table for primer sequences). A size marker (generated using primer Tel-cont' fwd / rev) was used as reference. The size-control probe is a specific region of chromosome II (positions 558490 to 559790) and detects two bands in the XhoI digested genomic DNA (2044 and 779bp long). Radiolabeling was performed following the protocol of the Roche High Prime DNA labeling KIT. Telomere length was measured with TelQuant [34], a VisualBasic6 program specifically developed for measuring telomere length in yeast and graphically displayed using Prism (GraphPad).

### dNTP level measurements

Measurements were performed as previously described [58]. S4 Table shows dNTP levels and ratios, as well as telomere length, for all mutants analyzed.

### Senescence experiments

Spore colonies of dissected diploids were re-suspended in water and diluted in 5 ml YPD to a final concentration of OD600 = 0.02. After 24 hours of growth at 30°C absorption at 600 nm was measured. Cultures were re-diluted to an OD600 = 0.02 in 5 ml YPD and inoculated for further 24 hours at 30°C. Cell samples have been stored daily for genomic DNA extraction and Southern teloblots. Population doublings (PD) were calculated as log2 (OD_600_24h/0.02). Graphs were created with the Prism (GraphPad) software package.

### Western blotting

Protein samples for Western blotting were prepared as described before [59]. Proteins were resolved by SDS-PAGE 10–15% acrylamide gels and transferred to nitrocellulose membranes, blocked with 5% milk in TBST and immunoblotted with the indicated antibodies. Detection was carried out using the ECL SuperSignal detection system (Thermo Scientific).

Rabbit polyclonal anti-Rnr1 (AS09 576), anti-Rnr3 (AS09 574), and anti-Sml1 (AS10 847) antibodies were produced by Agrisera, Sweden. For the detection of both Rnr4 and α-tubulin we used YL1/2 rat monoclonal antibodies (Sigma).

## Supporting information

S1 Fig *rnr3*Δ mutants show a wildtype telomere length. **The short telomeres of *rnr1***Δ ***crt1***Δ **mutants are not maintained by HDR. A)** Western blot analysis of RNR proteins. The upregulation of Rnr3/4 protein levels in *rnr1*Δ *crt1* mutants is comparable to that of *crt1*Δ mutants. **B)** Telomere length analysis by Southern blotting. The deletion of *RNR3* does not result in a short telomere phenotype in wildtype strains or *crt1*Δ mutants. Represented are biological replicates of the indicated strains after at least 200 generations. **C)** Telomere length analysis by Southern blotting. Following meiotic segregation heterozygous diploid *RNR1/rnr1*Δ *CRT1/crt1*Δ *RAD52/rad52*Δ mutants were dissected and the ability to maintain Y´ telomeres was analyzed in the indicated spore colonies after one over-night incubation in liquid culture (30 generations–G). *rnr1*Δ *crt1*Δ *rad52*Δ mutants show a short telomere length phenotype that resembles the one of *rnr1*Δ *crt1*Δ mutants indicating that Rad52-dependent HDR does neither contribute to the telomere maintenance nor to the telomere shortening of *rnr1*Δ *crt1*Δ mutants. Represented are biological replicates of the indicated strains. **D)** Telomere length analysis by Southern blotting. The deletion of *RAD52* does not exacerbate the short telomere phenotype of *rnr1*Δ *crt1*Δ mutants. Represented are biological replicates of the indicated strains after at least 200 generations.
(TIF)

S2 Fig The deletion of *CRT1* suppresses the lethality of *mec1*Δ to a similar extent than a deletion of *SML1*. **A)** Tetrad analysis of spores derived from *MEC1/mec1*Δ *SML1/sml1*Δ *RNR1/rnr1*Δ *CRT1/crt1*Δ heterozygote diploids. The genotypes of spore colonies were determined by replica plating on selective plates. The genotypes of dead spores were deduced from the genotypes of viable spores that derived from the same tetrad. All combinations between *mec1*Δ and *rnr1*Δ result in synthetic lethality (even in the absence of Sml1 and/or Crt1). A deletion of *CRT1* suppresses the lethality of *mec1*Δ similar to a deletion of *SML1*. Eight tetrads (1–8) have been analyzed by dissection. **B)** Telomere length has been analyzed as a function of cellular dGTP levels and dNTP levels. R-square tests have been performed to investigate the goodness of fit. n(dNTPs/length): wt (9/3), *rnr1*Δ (3/2), *crt1*Δ (2/2), *rnr1*Δ *crt1*Δ (3/3), *sml1*Δ *crt1*Δ (2/1), *mec1*Δ *sml1*Δ (2/3), *mec1*Δ *crt1*Δ (2/3), *mec1*Δ *sml1*Δ *crt1*Δ (2/3).
(TIF)

S3 Fig Replicative senescence is accelerated in the absence of Rnr1. **A)** Senescence curves reflecting the cell density reached after 24 hours of growth as a function of divisions. Single curves for *rnr1*Δ *crt1*Δ *est2*Δ and *crt1*Δ *est2*Δ mutants shown in Fig 3B. Two out of five biological replicates of *rnr1*Δ *crt1*Δ *est2*Δ were forming survivors already in the first re-dilution and therefore were not used to generate the average curve displayed in Fig 3B. The number of cell divisions the spore colony went through before the first dilution was estimated as 25 generations (starting point of the curves). **B)** Senescence curves were performed as described in Fig 3A. Telomerase positive *rnr1*Δ *crt1*Δ, *crt1*Δ and wild type cells did not show a loss of viability in serial dilutions. The number of cell divisions the spore colony went through before the first dilution has been estimated as 25 generations (starting point of the curves). Data is shown as mean +/- SEM (n = 3).
(TIF)

S4 Fig In the absence of Rnr1 and Crt1 telomeres do not become elongated when long *TLM* genes are deleted. Telomere length analysis by Southern blotting of the long *tlm* mutants *ogg1*Δ, *ppe1*Δ and *hsp104*Δ. Mutation of these genes cause telomere elongation when introduced in wild type cells but do not affect the short telomere length of *rnr1*Δ *crt1*Δ double mutants. Represented are biological replicates of the indicated strains after approximately 200 generations.
(TIF)

S5 Fig Telomere elongation in long-*tlm* mutants is promoted by Rnr1 upregulation through the checkpoint kinase Dun1. **A)** Telomere length analysis by Southern blotting. Deletion of the long *TLM* genes *MAK31*, *OGG1* and *HSP104* cause telomere elongation when introduced in a wild type or *elg1*Δ background. Deletion of these genes in Δ*dun1* mutants results in loss (*MAK31*) or reduction (*OGG1*, *HSP104*) of telomere elongation. Represented are biological replicates of the indicated strains after approximately 200 generations. **B)** Telomere length analysis by Southern blotting of cells expressing Cdc13-Est1 and Cdc13-Est2. Expression of the fusion proteins results in telomere elongation in *dun1*Δ *crt1*Δ mutants. Represented are biological replicates of the indicated strains after approximately 200 generations.
(TIF)

S1 Table Yeast strains used in this study. All strains were derived from the BY4741/2 (*MAT a/alpha his3*Δ*0 leu2*Δ*0 ura3*Δ*0*) background.
(PDF)

S2 Table Oligonucleotides used in this study. (TIF)

S3 Table Plasmids used in this study. (TIF)

S4 Table Raw data–Telomere length–dNTPs. (TIF)

## References

[1] Sfeir A, de Lange T (2012) Removal of shelterin reveals the telomere end-protection problem. Science 336: 593–597. doi: 10.1126/science.1218498 22556254 10.1126/science.1218498 PMC3477646

[2] Lingner J, Cooper JP, Cech TR (1995) Telomerase and DNA end replication: no longer a lagging strand problem? Science 269: 1533–1534. 7545310 10.1126/science.7545310

[3] O'Sullivan RJ, Karlseder J (2010) Telomeres: protecting chromosomes against genome instability. Nat Rev Mol Cell Biol 11: 171–181. doi: 10.1038/nrm2848 20125188 10.1038/nrm2848 PMC2842081

[4] Lundblad V, Szostak JW (1989) A mutant with a defect in telomere elongation leads to senescence in yeast. Cell 57: 633–643. 2655926 10.1016/0092-8674(89)90132-3

[5] Bodnar AG, Ouellette M, Frolkis M, Holt SE, Chiu CP, et al (1998) Extension of life-span by introduction of telomerase into normal human cells. Science 279: 349–352. 9454332 10.1126/science.279.5349.349

[6] Harley CB, Futcher AB, Greider CW (1990) Telomeres shorten during ageing of human fibroblasts. Nature 345: 458–460. doi: 10.1038/345458a0 2342578 10.1038/345458a0

[7] Kaul Z, Cesare AJ, Huschtscha LI, Neumann AA, Reddel RR (2012) Five dysfunctional telomeres predict onset of senescence in human cells. EMBO Rep 13: 52–59. 10.1038/embor.2011.227 PMC3246253 22157895

[8] Forsyth NR, Wright WE, Shay JW (2002) Telomerase and differentiation in multicellular organisms: turn it off, turn it on, and turn it off again. Differentiation 69: 188–197. doi: 10.1046/j.1432-0436.2002.690412.x 11841477 10.1046/j.1432-0436.2002.690412.x

[9] Hug N, Lingner J (2006) Telomere length homeostasis. Chromosoma 115: 413–425. doi: 10.1007/s00412-006-0067-3 16741708 10.1007/s00412-006-0067-3

[10] Nabetani A, Ishikawa F (2011) Alternative lengthening of telomeres pathway: recombination-mediated telomere maintenance mechanism in human cells. J Biochem 149: 5–14. doi: 10.1093/jb/mvq119 20937668 10.1093/jb/mvq119

[11] Cesare AJ, Reddel RR (2010) Alternative lengthening of telomeres: models, mechanisms and implications. Nat Rev Genet 11: 319–330. doi: 10.1038/nrg2763 20351727 10.1038/nrg2763

[12] Khadaroo B, Teixeira MT, Luciano P, Eckert-Boulet N, Germann SM, et al (2009) The DNA damage response at eroded telomeres and tethering to the nuclear pore complex. Nat Cell Biol 11: 980–987. doi: 10.1038/ncb1910 19597487 10.1038/ncb1910

[13] Lundblad V, Blackburn EH (1993) An alternative pathway for yeast telomere maintenance rescues est1- senescence. Cell 73: 347–360. 8477448 10.1016/0092-8674(93)90234-h

[14] Teng SC, Zakian VA (1999) Telomere-telomere recombination is an efficient bypass pathway for telomere maintenance in Saccharomyces cerevisiae. Mol Cell Biol 19: 8083–8093. 10567534 10.1128/mcb.19.12.8083 PMC84893

[15] Lydeard JR, Jain S, Yamaguchi M, Haber JE (2007) Break-induced replication and telomerase-independent telomere maintenance require Pol32. Nature 448: 820–823. doi: 10.1038/nature06047 17671506 10.1038/nature06047

[16] Askree SH, Yehuda T, Smolikov S, Gurevich R, Hawk J, et al (2004) A genome-wide screen for Saccharomyces cerevisiae deletion mutants that affect telomere length. Proc Natl Acad Sci U S A 101: 8658–8663. doi: 10.1073/pnas.0401263101 15161972 10.1073/pnas.0401263101 PMC423251

[17] Gatbonton T, Imbesi M, Nelson M, Akey JM, Ruderfer DM, et al (2006) Telomere length as a quantitative trait: genome-wide survey and genetic mapping of telomere length-control genes in yeast. PLoS Genet 2: e35 doi: 10.1371/journal.pgen.0020035 16552446 10.1371/journal.pgen.0020035 PMC1401499

[18] Ungar L, Yosef N, Sela Y, Sharan R, Ruppin E, et al (2009) A genome-wide screen for essential yeast genes that affect telomere length maintenance. Nucleic Acids Res 37: 3840–3849. doi: 10.1093/nar/gkp259 19386622 10.1093/nar/gkp259 PMC2709559

[19] Nordlund P, Reichard P (2006) Ribonucleotide reductases. Annu Rev Biochem 75: 681–706. doi: 10.1146/annurev.biochem.75.103004.142443 16756507 10.1146/annurev.biochem.75.103004.142443

[20] Guarino E, Salguero I, Kearsey SE (2014) Cellular regulation of ribonucleotide reductase in eukaryotes. Semin Cell Dev Biol 30: 97–103. doi: 10.1016/j.semcdb.2014.03.030 24704278 10.1016/j.semcdb.2014.03.030

[21] Sanvisens N, de Llanos R, Puig S (2013) Function and regulation of yeast ribonucleotide reductase: cell cycle, genotoxic stress, and iron bioavailability. Biomed J 36: 51–58. doi: 10.4103/2319-4170.110398 23644233 10.4103/2319-4170.110398

[22] Wang PJ, Chabes A, Casagrande R, Tian XC, Thelander L, et al (1997) Rnr4p, a novel ribonucleotide reductase small-subunit protein. Mol Cell Biol 17: 6114–6121. 9315671 10.1128/mcb.17.10.6114 PMC232461

[23] Sommerhalter M, Voegtli WC, Perlstein DL, Ge J, Stubbe J, et al (2004) Structures of the yeast ribonucleotide reductase Rnr2 and Rnr4 homodimers. Biochemistry 43: 7736–7742. doi: 10.1021/bi049510m 15196016 10.1021/bi049510m

[24] Zhao X, Chabes A, Domkin V, Thelander L, Rothstein R (2001) The ribonucleotide reductase inhibitor Sml1 is a new target of the Mec1/Rad53 kinase cascade during growth and in response to DNA damage. EMBO J 20: 3544–3553. doi: 10.1093/emboj/20.13.3544 11432841 10.1093/emboj/20.13.3544 PMC125510

[25] Andreson BL, Gupta A, Georgieva BP, Rothstein R (2010) The ribonucleotide reductase inhibitor, Sml1, is sequentially phosphorylated, ubiquitylated and degraded in response to DNA damage. Nucleic Acids Res 38: 6490–6501. doi: 10.1093/nar/gkq552 20566477 10.1093/nar/gkq552 PMC2965251

[26] Lee YD, Wang J, Stubbe J, Elledge SJ (2008) Dif1 is a DNA-damage-regulated facilitator of nuclear import for ribonucleotide reductase. Mol Cell 32: 70–80. doi: 10.1016/j.molcel.2008.08.018 18851834 10.1016/j.molcel.2008.08.018 PMC3245869

[27] Wu X, Huang M (2008) Dif1 controls subcellular localization of ribonucleotide reductase by mediating nuclear import of the R2 subunit. Mol Cell Biol 28: 7156–7167. doi: 10.1128/MCB.01388-08 18838542 10.1128/MCB.01388-08 PMC2593381

[28] Tsaponina O, Barsoum E, Astrom SU, Chabes A (2011) Ixr1 is required for the expression of the ribonucleotide reductase Rnr1 and maintenance of dNTP pools. PLoS Genet 7: e1002061 doi: 10.1371/journal.pgen.1002061 21573136 10.1371/journal.pgen.1002061 PMC3088718

[29] Elledge SJ, Davis RW (1990) Two genes differentially regulated in the cell cycle and by DNA-damaging agents encode alternative regulatory subunits of ribonucleotide reductase. Genes Dev 4: 740–751. 2199320 10.1101/gad.4.5.740

[30] Huang M, Zhou Z, Elledge SJ (1998) The DNA replication and damage checkpoint pathways induce transcription by inhibition of the Crt1 repressor. Cell 94: 595–605. 9741624 10.1016/s0092-8674(00)81601-3

[31] Domkin V, Thelander L, Chabes A (2002) Yeast DNA damage-inducible Rnr3 has a very low catalytic activity strongly stimulated after the formation of a cross-talking Rnr1/Rnr3 complex. J Biol Chem 277: 18574–18578. doi: 10.1074/jbc.M201553200 11893751 10.1074/jbc.M201553200

[32] Gupta A, Sharma S, Reichenbach P, Marjavaara L, Nilsson AK, et al (2013) Telomere length homeostasis responds to changes in intracellular dNTP pools. Genetics 193: 1095–1105. doi: 10.1534/genetics.112.149120 23335335 10.1534/genetics.112.149120 PMC3606089

[33] Zhao X, Muller EG, Rothstein R (1998) A suppressor of two essential checkpoint genes identifies a novel protein that negatively affects dNTP pools. Mol Cell 2: 329–340. 9774971 10.1016/s1097-2765(00)80277-4

[34] Rubinstein L, Ungar L, Harari Y, Babin V, Ben-Aroya S, et al (2014) Telomere length kinetics assay (TELKA) sorts the telomere length maintenance (tlm) mutants into functional groups. Nucleic Acids Res 42: 6314–6325. doi: 10.1093/nar/gku267 24728996 10.1093/nar/gku267 PMC4041441

[35] Claussin C, Chang M (2016) Multiple Rad52-Mediated Homology-Directed Repair Mechanisms Are Required to Prevent Telomere Attrition-Induced Senescence in Saccharomyces cerevisiae. PLoS Genet 12: e1006176 doi: 10.1371/journal.pgen.1006176 27428329 10.1371/journal.pgen.1006176 PMC4948829

[36] Zhao X, Georgieva B, Chabes A, Domkin V, Ippel JH, et al (2000) Mutational and structural analyses of the ribonucleotide reductase inhibitor Sml1 define its Rnr1 interaction domain whose inactivation allows suppression of mec1 and rad53 lethality. Mol Cell Biol 20: 9076–9083. 11074005 10.1128/mcb.20.23.9076-9083.2000 PMC86560

[37] Longhese MP, Paciotti V, Neecke H, Lucchini G (2000) Checkpoint proteins influence telomeric silencing and length maintenance in budding yeast. Genetics 155: 1577–1591. 10924458 10.1093/genetics/155.4.1577 PMC1461196

[38] Jay KA, Smith DL, Blackburn EH (2016) Early Loss of Telomerase Action in Yeast Creates a Dependence on the DNA Damage Response Adaptor Proteins. Mol Cell Biol 36: 1908–1919. doi: 10.1128/MCB.00943-15 27161319 10.1128/MCB.00943-15 PMC4936065

[39] Boule JB, Vega LR, Zakian VA (2005) The yeast Pif1p helicase removes telomerase from telomeric DNA. Nature 438: 57–61. doi: 10.1038/nature04091 16121131 10.1038/nature04091

[40] Gazy I, Liefshitz B, Parnas O, Kupiec M (2015) Elg1, a central player in genome stability. Mutat Res Rev Mutat Res 763: 267–279. doi: 10.1016/j.mrrev.2014.11.007 25795125 10.1016/j.mrrev.2014.11.007

[41] Smolikov S, Mazor Y, Krauskopf A (2004) ELG1, a regulator of genome stability, has a role in telomere length regulation and in silencing. Proc Natl Acad Sci U S A 101: 1656–1661. doi: 10.1073/pnas.0307796100 14745004 10.1073/pnas.0307796100 PMC341813

[42] Banerjee S, Myung K (2004) Increased genome instability and telomere length in the elg1-deficient Saccharomyces cerevisiae mutant are regulated by S-phase checkpoints. Eukaryot Cell 3: 1557–1566. doi: 10.1128/EC.3.6.1557-1566.2004 15590829 10.1128/EC.3.6.1557-1566.2004 PMC539025

[43] Evans SK, Lundblad V (1999) Est1 and Cdc13 as comediators of telomerase access. Science 286: 117–120. 10506558 10.1126/science.286.5437.117

[44] Fasullo M, Tsaponina O, Sun M, Chabes A (2010) Elevated dNTP levels suppress hyper-recombination in Saccharomyces cerevisiae S-phase checkpoint mutants. Nucleic Acids Res 38: 1195–1203. doi: 10.1093/nar/gkp1064 19965764 10.1093/nar/gkp1064 PMC2831302

[45] Yao R, Zhang Z, An X, Bucci B, Perlstein DL, et al (2003) Subcellular localization of yeast ribonucleotide reductase regulated by the DNA replication and damage checkpoint pathways. Proc Natl Acad Sci U S A 100: 6628–6633. doi: 10.1073/pnas.1131932100 12732713 10.1073/pnas.1131932100 PMC164498

[46] Tkach JM, Yimit A, Lee AY, Riffle M, Costanzo M, et al (2012) Dissecting DNA damage response pathways by analysing protein localization and abundance changes during DNA replication stress. Nat Cell Biol 14: 966–976. doi: 10.1038/ncb2549 22842922 10.1038/ncb2549 PMC3434236

[47] Niida H, Katsuno Y, Sengoku M, Shimada M, Yukawa M, et al (2010) Essential role of Tip60-dependent recruitment of ribonucleotide reductase at DNA damage sites in DNA repair during G1 phase. Genes Dev 24: 333–338. doi: 10.1101/gad.1863810 20159953 10.1101/gad.1863810 PMC2816732

[48] Lue NF (2004) Adding to the ends: what makes telomerase processive and how important is it? Bioessays 26: 955–962. doi: 10.1002/bies.20093 15351966 10.1002/bies.20093

[49] Bosoy D, Lue NF (2004) Yeast telomerase is capable of limited repeat addition processivity. Nucleic Acids Res 32: 93–101. doi: 10.1093/nar/gkg943 14704347 10.1093/nar/gkg943 PMC373262

[50] Hardy CD, Schultz CS, Collins K (2001) Requirements for the dGTP-dependent repeat addition processivity of recombinant Tetrahymena telomerase. J Biol Chem 276: 4863–4871. doi: 10.1074/jbc.M005158200 11096070 10.1074/jbc.M005158200

[51] Peng Y, Mian IS, Lue NF (2001) Analysis of telomerase processivity: mechanistic similarity to HIV-1 reverse transcriptase and role in telomere maintenance. Mol Cell 7: 1201–1211. 11430823 10.1016/s1097-2765(01)00268-4

[52] Maine IP, Chen SF, Windle B (1999) Effect of dGTP concentration on human and CHO telomerase. Biochemistry 38: 15325–15332. 10563818 10.1021/bi991596+

[53] Sun D, Lopez-Guajardo CC, Quada J, Hurley LH, Von Hoff DD (1999) Regulation of catalytic activity and processivity of human telomerase. Biochemistry 38: 4037–4044. doi: 10.1021/bi982249n 10194316 10.1021/bi982249n

[54] Grandin N, Bailly A, Charbonneau M (2005) Activation of Mrc1, a mediator of the replication checkpoint, by telomere erosion. Biol Cell 97: 799–814. doi: 10.1042/BC20040526 15760303 10.1042/BC20040526

[55] Morafraile EC, Diffley JF, Tercero JA, Segurado M (2015) Checkpoint-dependent RNR induction promotes fork restart after replicative stress. Sci Rep 5: 7886 doi: 10.1038/srep07886 25601385 10.1038/srep07886 PMC4298733

[56] Aye Y, Li M, Long MJ, Weiss RS (2015) Ribonucleotide reductase and cancer: biological mechanisms and targeted therapies. Oncogene 34: 2011–2021. doi: 10.1038/onc.2014.155 24909171 10.1038/onc.2014.155

[57] Tong AS, Stern JL, Sfeir A, Kartawinata M, de Lange T, et al (2015) ATM and ATR Signaling Regulate the Recruitment of Human Telomerase to Telomeres. Cell Rep 13: 1633–1646. doi: 10.1016/j.celrep.2015.10.041 26586433 10.1016/j.celrep.2015.10.041 PMC4662887

[58] Jia S, Marjavaara L, Buckland R, Sharma S, Chabes A (2015) Determination of deoxyribonucleoside triphosphate concentrations in yeast cells by strong anion-exchange high-performance liquid chromatography coupled with ultraviolet detection. Methods Mol Biol 1300: 113–121. doi: 10.1007/978-1-4939-2596-4_8 25916709 10.1007/978-1-4939-2596-4_8

[59] Salomon D, Sessa G (2010) Identification of growth inhibition phenotypes induced by expression of bacterial type III effectors in yeast. J Vis Exp. 10.3791/1865 PMC3168205 20354502

