# Supplementary figures and images for: Rnr1, but not Rnr3, facilitates the sustained telomerase-dependent elongation of telomeres
Source: PLoS Genet. 2017 Oct 25;13(10):e1007082. doi: 10.1371/journal.pgen.1007082 (PMC5673236; doi:10.1371/journal.pgen.1007082)

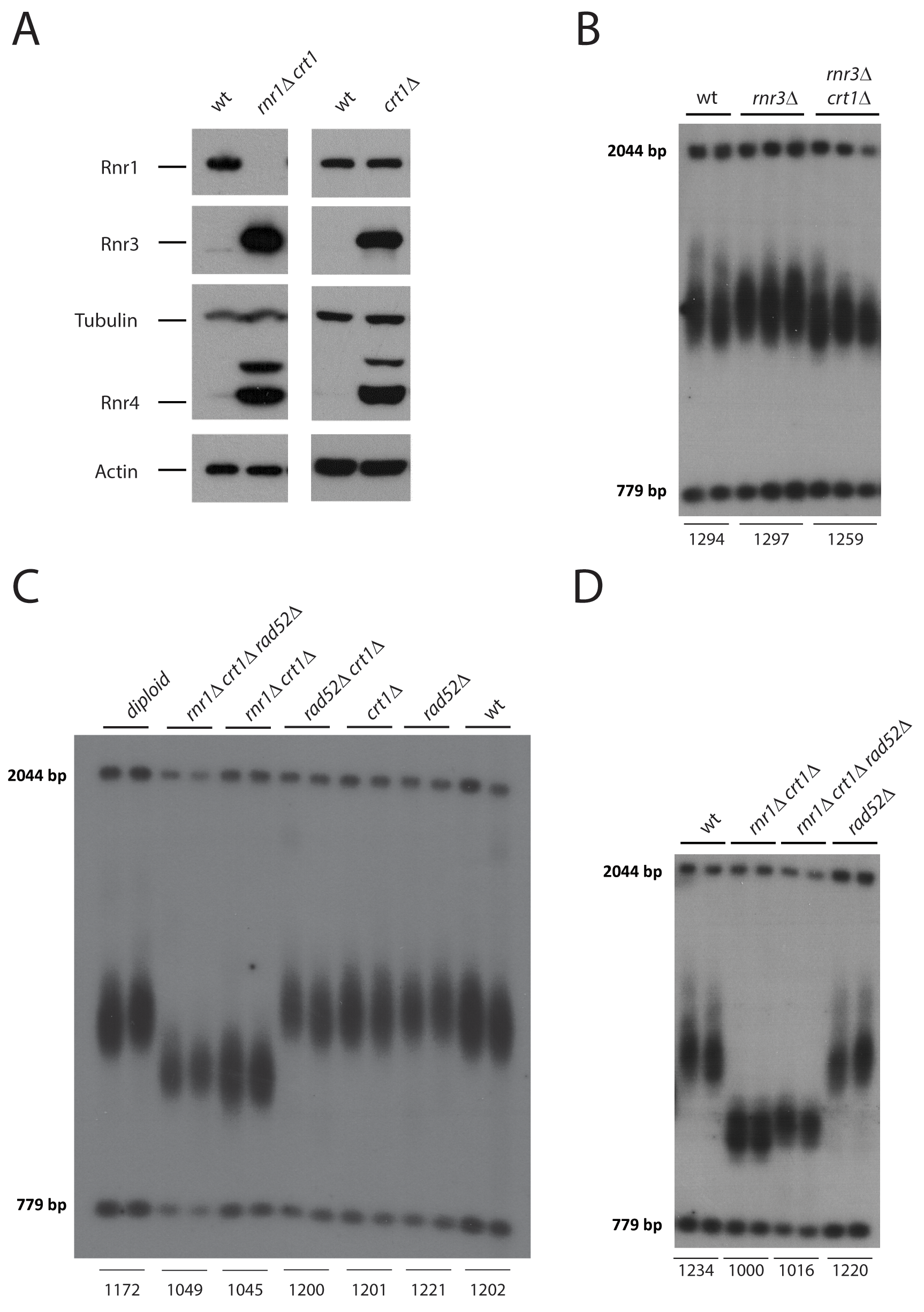

Supplement: S1 Fig — The short telomeres of rnr1Δ crt1Δ mutants are not maintained by HDR. A) Western blot analysis of RNR proteins. The upregulation of Rnr3/4 protein levels in rnr1Δ crt1 mutants is comparable to that of crt1Δ mutants. B) Telomere length analysis by Southern blotting. The deletion of RNR3 does not result in a short telomere phenotype in wildtype strains or crt1Δ mutants. Represented are biological replicates of the indicated strains after at least 200 generations. C) Telomere length analysis by Southern blotting. Following meiotic segregation heterozygous diploid RNR1/rnr1Δ CRT1/crt1Δ RAD52/rad52Δ mutants were dissected and the ability to maintain Y´ telomeres was analyzed in the indicated spore colonies after one over-night incubation in liquid culture (30 generations–G). rnr1Δ crt1Δ rad52Δ mutants show a short telomere length phenotype that resembles the one of rnr1Δ crt1Δ mutants indicating that Rad52-dependent HDR does neither contribute to the telomere maintenance nor to the telomere shortening of rnr1Δ crt1Δ mutants. Represented are biological replicates of the indicated strains. D) Telomere length analysis by Southern blotting. The deletion of RAD52 does not exacerbate the short telomere phenotype of rnr1Δ crt1Δ mutants. Represented are biological replicates of the indicated strains after at least 200 generations. (TIF) [file pgen.1007082.s001.tif]

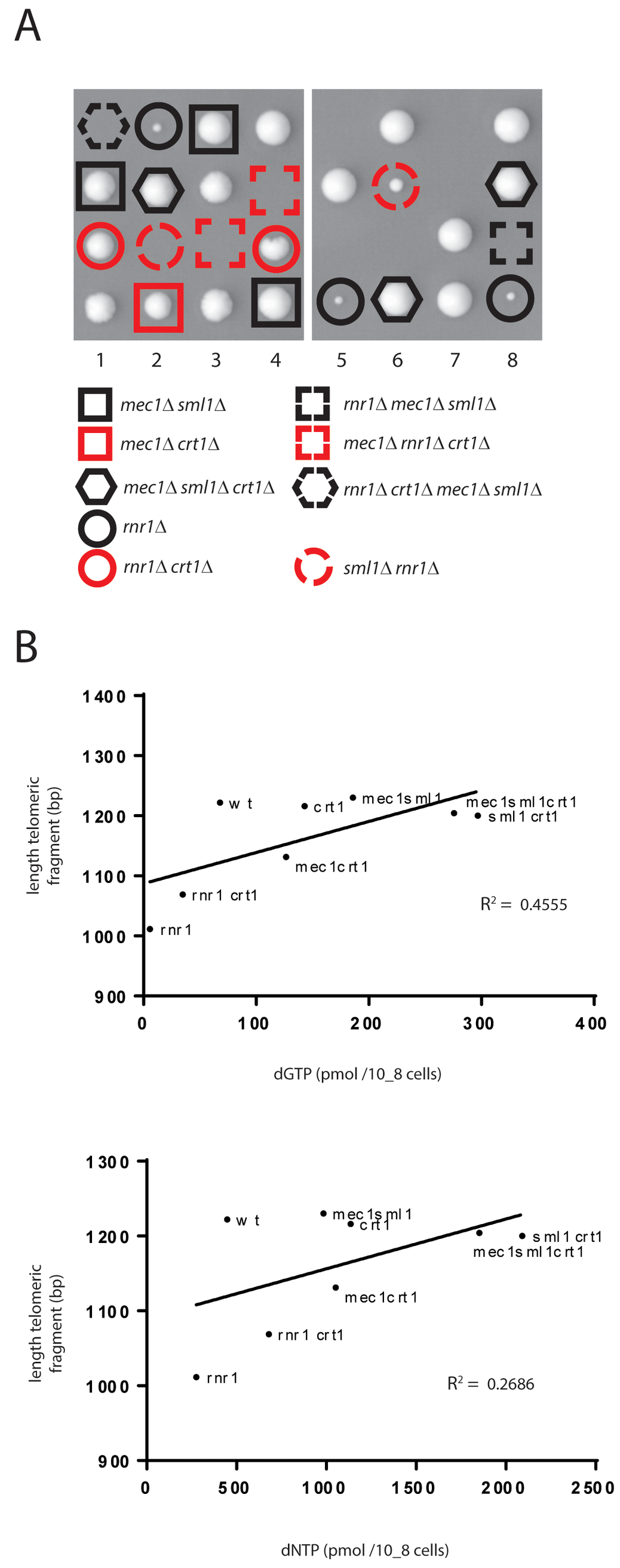

Supplement: S2 Fig — A) Tetrad analysis of spores derived from MEC1/mec1Δ SML1/sml1Δ RNR1/rnr1Δ CRT1/crt1Δ heterozygote diploids. The genotypes of spore colonies were determined by replica plating on selective plates. The genotypes of dead spores were deduced from the genotypes of viable spores that derived from the same tetrad. All combinations between mec1Δ and rnr1Δ result in synthetic lethality (even in the absence of Sml1 and/or Crt1). A deletion of CRT1 suppresses the lethality of mec1Δ similar to a deletion of SML1. Eight tetrads (1–8) have been analyzed by dissection. B) Telomere length has been analyzed as a function of cellular dGTP levels and dNTP levels. R-square tests have been performed to investigate the goodness of fit. n(dNTPs/length): wt (9/3), rnr1Δ (3/2), crt1Δ (2/2), rnr1Δ crt1Δ (3/3), sml1Δ crt1Δ (2/1), mec1Δ sml1Δ (2/3), mec1Δ crt1Δ (2/3), mec1Δ sml1Δ crt1Δ (2/3). (TIF) [file pgen.1007082.s002.tif]

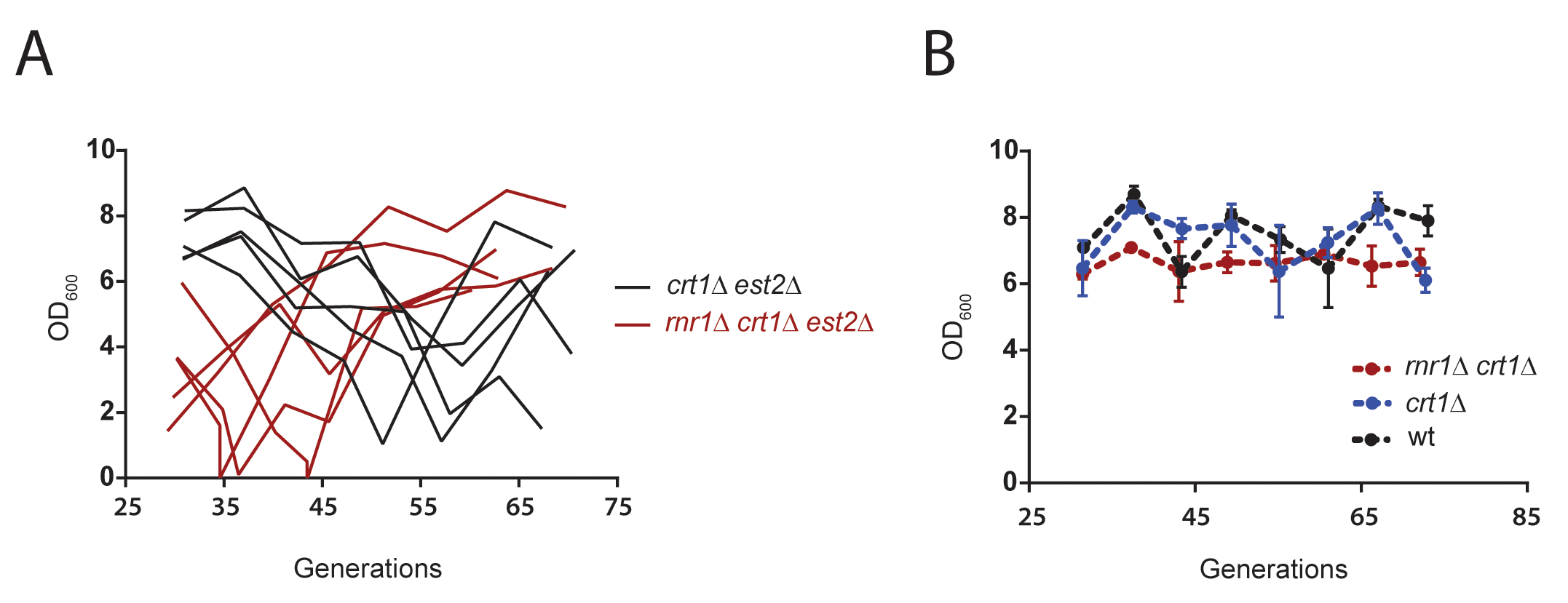

Supplement: S3 Fig — A) Senescence curves reflecting the cell density reached after 24 hours of growth as a function of divisions. Single curves for rnr1Δ crt1Δ est2Δ and crt1Δ est2Δ mutants shown in Fig 3B. Two out of five biological replicates of rnr1Δ crt1Δ est2Δ were forming survivors already in the first re-dilution and therefore were not used to generate the average curve displayed in Fig 3B. The number of cell divisions the spore colony went through before the first dilution was estimated as 25 generations (starting point of the curves). B) Senescence curves were performed as described in Fig 3A. Telomerase positive rnr1Δ crt1Δ, crt1Δ and wild type cells did not show a loss of viability in serial dilutions. The number of cell divisions the spore colony went through before the first dilution has been estimated as 25 generations (starting point of the curves). Data is shown as mean +/- SEM (n = 3). (TIF) [file pgen.1007082.s003.tif]

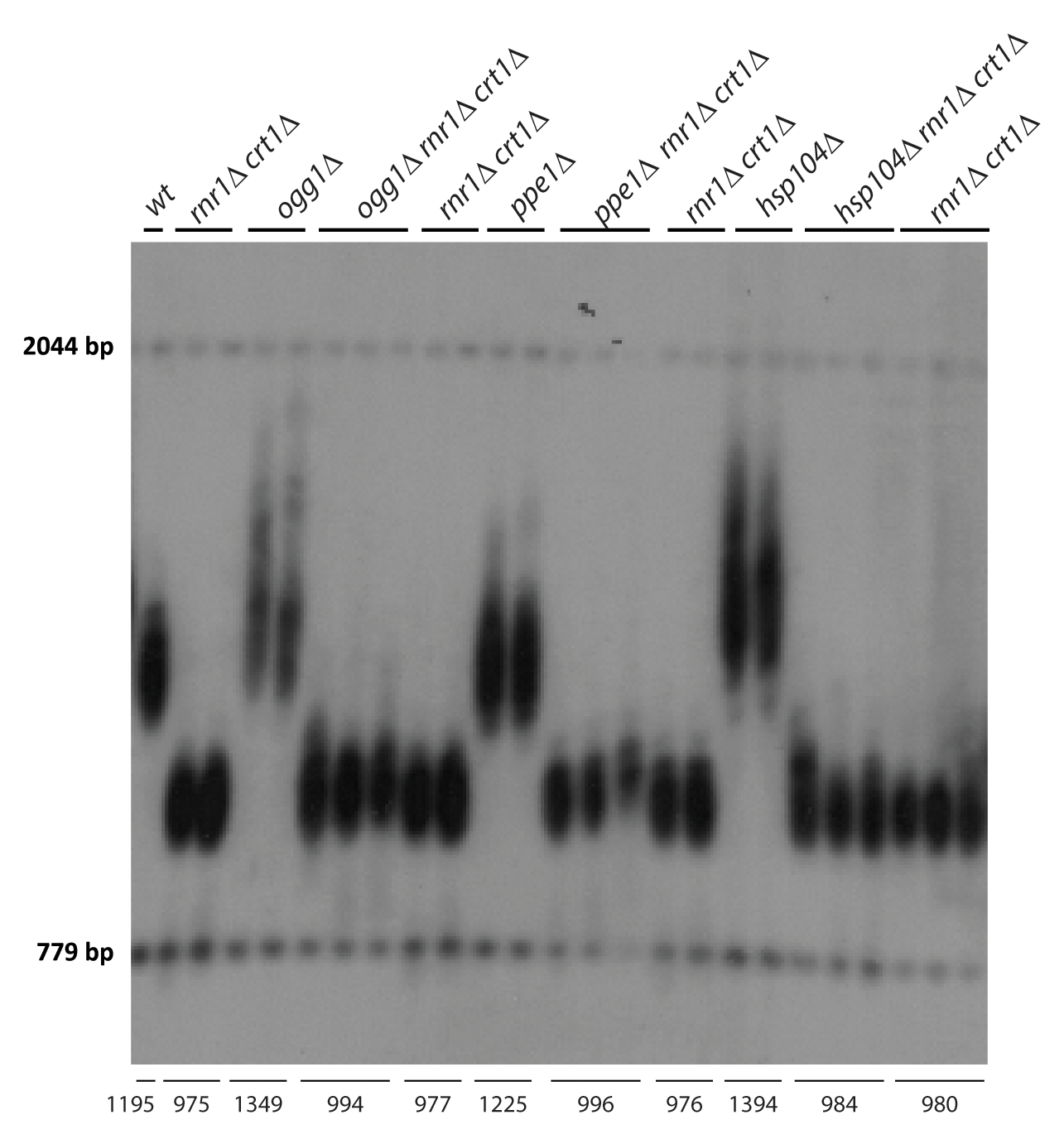

Supplement: S4 Fig — Telomere length analysis by Southern blotting of the long tlm mutants ogg1Δ, ppe1Δ and hsp104Δ. Mutation of these genes cause telomere elongation when introduced in wild type cells but do not affect the short telomere length of rnr1Δ crt1Δ double mutants. Represented are biological replicates of the indicated strains after approximately 200 generations. (TIF) [file pgen.1007082.s004.tif]

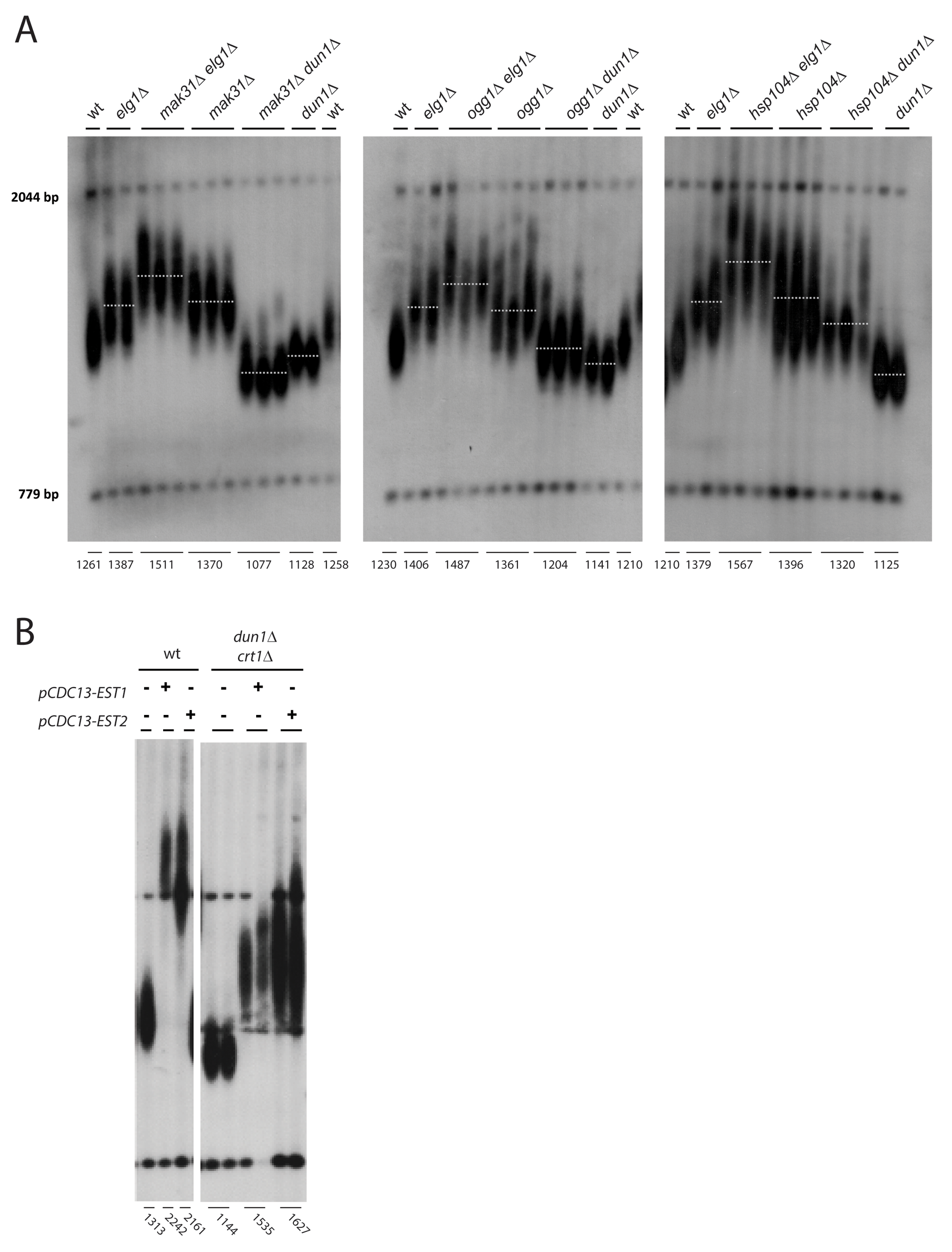

Supplement: S5 Fig — A) Telomere length analysis by Southern blotting. Deletion of the long TLM genes MAK31, OGG1 and HSP104 cause telomere elongation when introduced in a wild type or elg1Δ background. Deletion of these genes in Δdun1 mutants results in loss (MAK31) or reduction (OGG1, HSP104) of telomere elongation. Represented are biological replicates of the indicated strains after approximately 200 generations. B) Telomere length analysis by Southern blotting of cells expressing Cdc13-Est1 and Cdc13-Est2. Expression of the fusion proteins results in telomere elongation in dun1Δ crt1Δ mutants. Represented are biological replicates of the indicated strains after approximately 200 generations. (TIF) [file pgen.1007082.s005.tif]

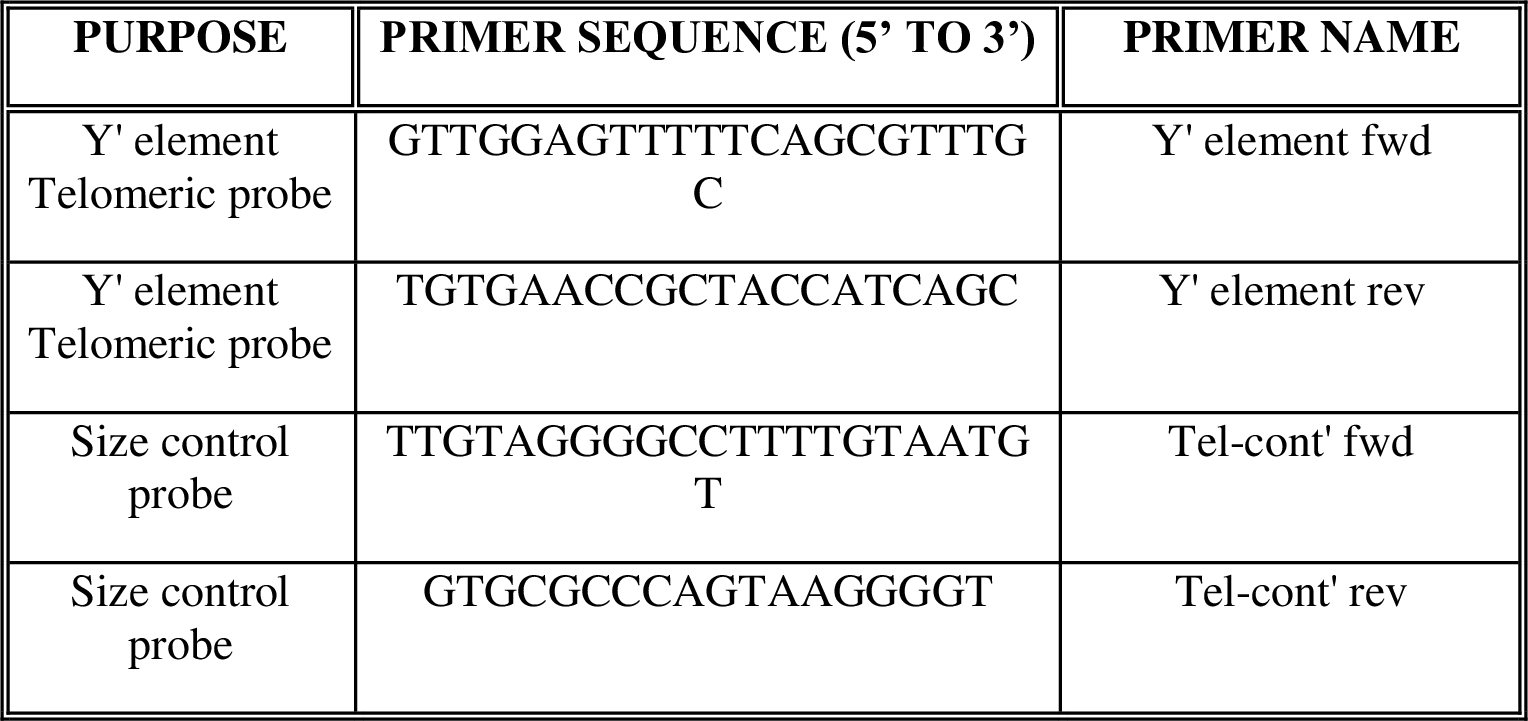

Supplement: S2 Table — (TIF) [file pgen.1007082.s007.tif]

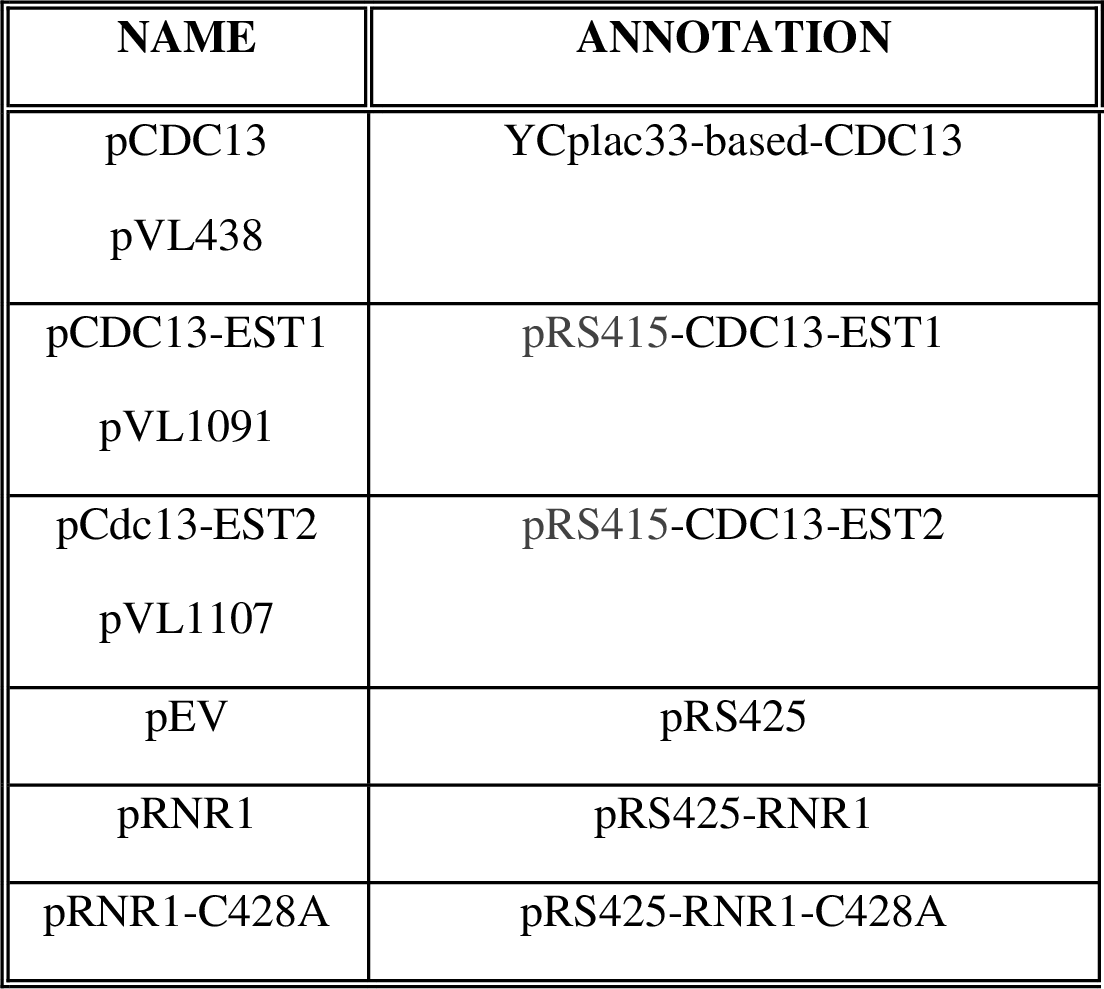

Supplement: S3 Table — (TIF) [file pgen.1007082.s008.tif]

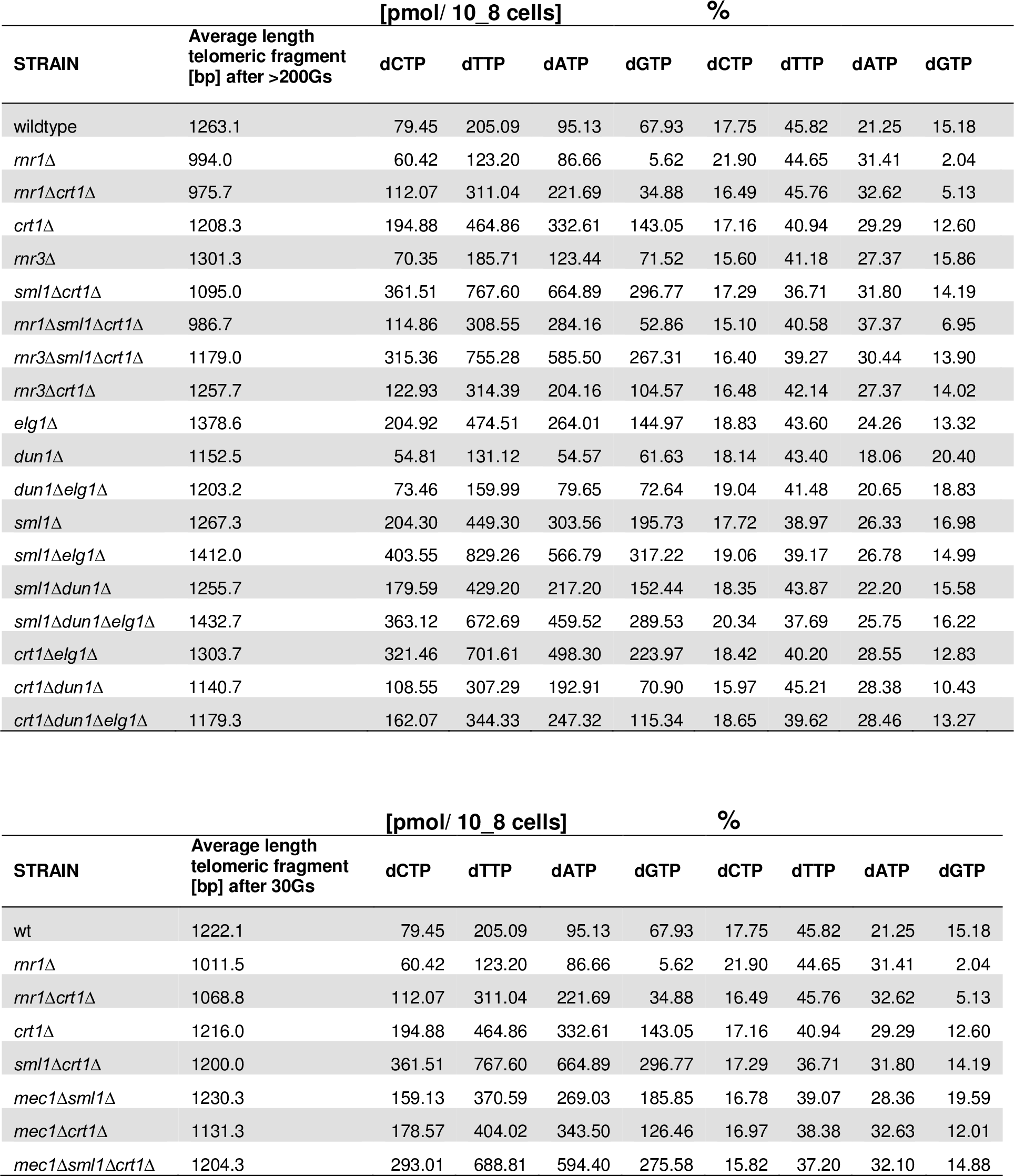

Supplement: S4 Table — (TIF) [file pgen.1007082.s009.tif]
