# Supplementary material for: Rnr1, but not Rnr3, facilitates the sustained telomerase-dependent elongation of telomeres
Source: PLoS Genet. 2017 Oct 25;13(10):e1007082. doi: 10.1371/journal.pgen.1007082 (PMC5673236; doi:10.1371/journal.pgen.1007082)
Supplement: S1 Table — All strains were derived from the BY4741/2 (MAT a/alpha his3Δ0 leu2Δ0 ura3Δ0) background. (PDF) [file pgen.1007082.s006.pdf]

rnr1Δ crt1	*rnr1::KanMX crt1 809T>A*
rnr1Δ	*rnr1::HYG*
crt1Δ	*crt1::HIS3 and crt1::KanMX*
rnr1Δcrt1Δ	*rnr1::HYG crt1::HIS3*
rnr1Δ crt1Δ sml1Δ	*rnr1::Ura crt1::HIS sml1::KanMX*
sml1Δ	*sml1::KanMX*
rnr1Δ crt1 – pEV	*rnr1::KanMX crt1 809T>A pEV*
rnr1Δ crt1 – pRNR1	*rnr1::KanMX crt1 809T>A pRNR1*
rnr1Δ crt1-pRNR1-C428A	*rnr1::KanMX crt1 809T>A pRNR1-C428A*
RNR1/rnr1Δ CRT1/crt1Δ	*RNR1/ rnr1::HYG CRT1/crt1::HIS3*
elg1Δ	*elg1::HYG*
rnr1Δ crt1 elg1Δ	*rnr1::KanMX crt1 809T>A elg1::HYG*
pif1Δ	*pif1::KanMX*
rnr1Δ crt1Δ pif1Δ	*rnr1::HYG pif1::KanMX crt1::HIS3*
pif1Δ crt1Δ	*crt1::HIS3 pif1:: KanMX*
wild type +Cdc13	*+ pCDC13*
wild type +Cdc13-Est1	*+ pCDC13-EST1*
wild type +Cdc13-Est2	*+ pCDC13-EST2*
rnr1Δ crt1 + Cdc13-Est1	*rnr1::KanMX crt1 809T>A + pCDC13-EST1*
rnr1Δ crt1 + Cdc13-Est2	*rnr1::KanMX crt1 809T>A + pCDC13-EST2*
Rnr1/rnr1Δ CRT1/crt1Δ EST2/est2Δ	*RNR1/rnr1::HYG CRT1/crt1::HIS3 EST2/est2::URA3*
rnr1Δ crt1Δ est2Δ	*rnr1::HYG crt1::HIS3 est2::URA*
crt1Δ est2Δ	*crt1::HIS3 est2::URA*
est2Δ	*est2::URA*
sml1Δ elg1Δ	*sml1::HIS3 elg1::HYG*

dun1Δ	*dun1::KanMX*
sml1Δ dun1Δ	*dun1::URA3 sml1::HIS3*
dun1Δ elg1Δ	*dun1::KanMX elg1::HYG*
sml1Δ dun1Δ elg1Δ	*dun1::KanMX elg1::HYG sml1::HIS3*
elg1Δ crt1Δ	*crt1:KanMX elg1::HYG*
dun1Δ crt1Δ	*crt1::KanMX dun1::URA3*
crt1Δ dun1Δ elg1Δ	*crt1::KanMX dun1::URA3 elg1::HYG*
MEC1/mec1Δ SML1/sml1Δ RNR1/rnr1Δ CRT1/crt1Δ	*RNR1/rnr1::URA3 CRT1/crt1::HIS3 Mec1/mec1::HYG SML1/sml1::KanMX*
mec1Δ sml1Δ	*mec1::HYG sml1::KanMX*
mec1Δ crt1Δ	*crt1::HIS3 mec1::HYG*
sml1Δ crt1Δ	*crt1::HIS3 sml1::KanMX*
mec1Δ sml1Δ crt1Δ	*crt1::HIS3 mec1::HYG sml1::KanMX*
ogg1Δ	*ogg1::KanMX*
ogg1Δ rnr1Δ crt1	*rnr1::HYG crt1 809T>A ogg1::KanMX*
ogg1Δ elg1Δ	*ogg1::KanMX elg1::HYG*
ogg1Δ dun1Δ	*ogg1::KanMX dun1::URA3*
ppe1Δ	*ppe1::KanMX*
ppe1Δ rnr1Δ crt1	*rnr1::HYG crt1 809T>A ppe1::KanMX*
hsp104Δ	*hsp104::KanMX*
hsp104Δ rnr1Δ crt1	*rnr1::HYG crt1 809T>A hsp104::KanMX*
hsp104Δ elg1Δ	*hsp104::KanMX elg1::HYG*
hsp104Δ dun1Δ	*hsp104::KanMX dun1::URA3*
RAD52/rad52Δ RNR1/rnr1Δ CRT1/crt1Δ	*RAD52/rad52::KanMX RNR1/rnr1::HYG CRT1/crt1::HIS3*
rnr1Δ crt1Δ rad52Δ	*rad52::KanMX rnr1::HYG crt1::HIS3*
crt1Δ rad52Δ	*rad52::KanMX crt1::HIS3*

rad52Δ	*rad52::KanMX*
mak31Δ	*mak31::KanMX*
mak31Δ elg1Δ	*mak31::KanMX elg1::HYG*
mak31Δ dun1Δ	*mak31::KanMX dun1::URA3*
rnr3Δ	*rnr3::KanMX*
rnr3Δ crt1Δ	*rnr3::KanMX crt1::HIS3*
rnr3Δ crt1Δ sml1Δ	*rnr3::KanMX sml1::HYG crt1::HIS3*
